# Partial Niche Partitioning in Three Sympatric Gull Species Through Foraging Areas and Habitat Selection

**DOI:** 10.1002/ece3.71577

**Published:** 2025-07-01

**Authors:** Nina J. O'Hanlon, Gary D. Clewley, Daniel T. Johnston, Chris B. Thaxter, Samuel Langlois Lopez, Lucy R. Quinn, Philipp H. Boersch‐Supan, Elizabeth A. Masden, Francis Daunt, Jared Wilson, Niall H. K. Burton, Elizabeth M. Humphreys

**Affiliations:** ^1^ BTO Scotland, Beta Centre (Unit 15) Stirling University Innovation Park Stirling UK; ^2^ British Trust for Ornithology Norfolk UK; ^3^ Environmental Research Institute, UHI North, West and Hebrides University of the Highlands and Islands Thurso UK; ^4^ NatureScot Inverness UK; ^5^ UK Centre for Ecology & Hydrology Midlothian UK; ^6^ Marine Directorate Aberdeen UK

**Keywords:** anthropogenic resources, competition, GPS tracking, resource selection, seabirds, specialisation

## Abstract

Anthropogenic habitat change is having a detrimental impact on biodiversity worldwide, altering the foraging behaviour and population dynamics of many species. Generalist species often adapt by broadening their resource use and/or exploiting human‐modified environments. However, habitat changes that reduce the availability of good quality resources can lead to increased interspecific competition among sympatric species and increased conflict with human activities. We investigated the breeding season foraging ecology of three sympatric gull species, Lesser Black‐backed ( *Larus fuscus* ), Herring ( *Larus argentatus* ) and Great Black‐backed Gulls ( *Larus marinus* ), from the same colony in Scotland. Using GPS tracking data, we analysed foraging ranges, spatial distributions and habitat preferences to determine the extent of the gulls' niche partitioning and use of human‐modified landscapes. Our findings revealed considerable overlap in resource use between species. However, species‐level differences in spatial distributions and habitat selection demonstrated partial niche partitioning. Lesser Black‐backed Gulls had significantly larger foraging ranges than Herring and Great Black‐backed Gulls, indicating spatial segregation. Herring and Great Black‐backed Gulls strongly selected for landfill and coastal habitats. Lesser Black‐backed Gulls also selected for these habitats but primarily used agricultural areas. Individual‐level analysis revealed that most species‐level selection for urban, landfill and harbour habitats was driven by a subset of individuals. The observed limited niche partitioning indicates that further habitat loss or degradation could negatively impact all three gull species unless the extent of niche partitioning changes. Given that most habitats used were linked to human activities, further anthropogenic change may displace gulls from preferred foraging areas, increasing competition for limited resources and exacerbating conflicts with human activities in alternative habitats. By simultaneously tracking sympatric species, we can better understand how shifts in resource availability may impact interspecific competition and interactions with human activities to help inform management actions and mitigate conflict with humans, particularly around licensed control.

## Graphical abstract

Our study analysed GPS data from three sympatric, generalist gull species in the Firth of Forth, Scotland, to investigate niche partitioning, focusing on spatial distributions and resource use, including population‐ and individual‐level habitat preferences. Whilst the gulls showed overlap in habitat use, subtle niche partitioning did occur, including spatial segregation in core foraging areas and varying selection for landfill, coastal and agricultural habitats. These findings highlight the potential ecological and socio‐economic consequences of habitat shifts driven by anthropogenic pressures, including increased conflict with human activities and impacts on gull population dynamics.

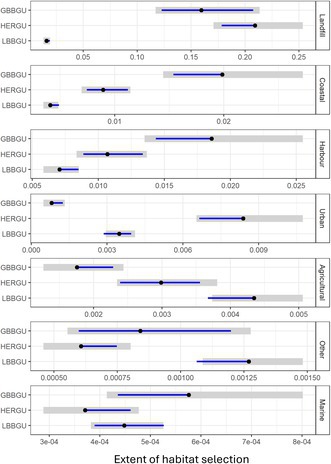

## 1 Introduction

The combined impact of anthropogenic driven climate change with extensive habitat loss, fragmentation and modification is having a detrimental effect on marine and terrestrial ecosystems globally with adverse consequences on biodiversity (Mantyka‐pringle et al. 2012; Maxwell et al. 2016, 2013). The extent to which species are affected can be influenced by their ability to adapt to changing environments (Christian et al. 2009; Clavel et al. 2011; Devictor et al. 2008). In resource‐limited environments, niche partitioning, where species partition space, time and/or resources, can allow species to coexist through reducing interspecific competition (MacArthur and Levins 1967; Navarro et al. 2013; Schoener 1974). As resource availability declines, for example through anthropogenic habitat loss or degradation, niche overlap between species is expected to decrease to reduce interspecific competition (Schoener 1982). Paradoxically, trophic niche widths are expected to expand as species consume more suboptimal resources (MacArthur and Pianka 1966). This typically results in generalist species becoming more dominant at the expense of specialists (Pagani‐Núñez et al. 2022; Sol et al. 2020); especially generalist species that benefit from predictable foraging and safe breeding opportunities provided by anthropogenic habitat change (Jokimaki and Suhonen 1993; McKinney and Lockwood 1999). However, even for adaptable, generalist species, habitat changes leading to the use of alternative, poorer quality resources, especially where competition for high‐quality habitats increases, can adversely impact populations (Colles et al. 2009).

Many species within the gull (Laridae) family are opportunistic foragers which have adapted from more traditional foraging behaviour, for example, searching for macro‐invertebrates at coastal and inland habitats and hunting fish at sea, to exploiting anthropogenic resources from a range of habitats and human activities (Belant et al. 1998; Duhem et al. 2008; Frixione et al. 2023; Ramos et al. 2012; Spelt et al. 2019). This flexibility alongside changes in the availability of traditional food sources has led several gull species to broaden their potential niche width and come into increased contact with humans or human activities. This is particularly the case when gulls take advantage of foraging opportunities associated with agriculture, including crops and livestock farming (Isaksson et al. 2016; Kubetzki and Garthe 2003), fishery discards and landings (Isaksson et al. 2016; Kubetzki and Garthe 2003), landfill sites (Belant et al. 1998) and urban areas (Shaffer et al. 2017; Spelt et al. 2019). Exploitation of such dependable anthropogenic food sources has also been linked to increased populations of some gull species, especially around urban and industrial areas, further increasing the likelihood that gulls and humans will come into contact (Duhem et al. 2008; Pons 1992; Verbeek 1977). Consequently, concern has grown around the potential for gull‐driven disease transmission and contamination of water bodies, as well as the nuisance associated with noise, faeces and perceived aggression (Ahlstrom et al. 2020; Belant 1997; Cockerham et al. 2019; Navarro et al. 2019; Rock 2005; Young et al. 2016). Furthermore, conflicts can occur where gulls are observed to negatively impact the populations of wildlife targeted for conservation, mainly though predation effects (Donehower and Bird 2008; Langlois Lopez, Clewley, et al. 2023). Interactions between gulls and human activities may also have negative consequences on the gulls themselves, for example, through increased mortality due to disease when foraging at landfill sites (Ortiz and Smith 1994), accidental bycatch when scavenging at fishing vessels (Žydelis et al. 2013) or increased collision risk when foraging inland or at sea around wind energy developments (Furness et al. 2013; Thaxter et al. 2019).

Given the wide range of foraging habitats that gulls exploit within both terrestrial and marine environments, they provide a useful case study to explore niche partitioning among sympatric, opportunistic species across heterogeneous, human‐modified landscapes. Although gulls are typically generalist foragers at the species level, individuals are often specialists but will readily switch between resources in response to their availability (Cimino et al. 2022; Davis 1975; Maynard and Ronconi 2018; McCleary and Sibly 1986). Foraging specialisation can have strong fitness benefits to individuals through reduced foraging effort and improved breeding success (Bolnick et al. 2003; van den Bosch et al. 2019). Most studies looking at habitat specialisation and competition within generalist species, including gulls, focus on single species and therefore intraspecific competition (Corman et al. 2016; Grémillet et al. 2004; Lee et al. 2021; Shaffer et al. 2017). However, interspecific competition is also a key driver of foraging decisions, often leading to niche and dietary partitioning between species (Estévanez and Aparicio 2019; Furness et al. 1992; Noordhuis and Spaans 1992; Calado et al. 2017; Rome and Ellis 2004; Ronconi et al. 2014). By characterising the foraging ecology of sympatric gull species from the same colony, including their foraging range, spatial distribution and habitat selection, we can identify any variation in where these species, or individuals within them, forage across human‐modified landscapes, and whether this results in variation in the extent to which they interact, and potential conflict, with human activities.

The use of modern biologging technology, particularly global positioning system (GPS) tracking, has enabled fine‐scale data on species space and habitat use to be collected from multiple individuals, including gulls (Bouten et al. 2013; Burger and Shaffer 2008). Using such data to determine foraging distributions and habitat selection provides a useful approach to understanding niche partitioning within and between species (Manly et al. 2002). Such analysis can identify the specific resources that different species or individuals prefer, thus indicating whether they may be partitioning resources and avoiding competition (Kazama et al. 2018; Tyson et al. 2015; van den Bosch et al. 2019), including those associated with human activities.

Here, we determine the habitat selection, at the population and individual level, of three sympatric large gull species (Great Black‐backed Gull *Larus marinus* , Herring Gull *Larus argentatus* and Lesser Black‐backed Gull *Larus fuscus* ). In north‐west Europe, these three species often occur in close association during the breeding season, including within mixed colonies (Grant et al. 2013; Outram and Steel 2021; Sellers and Shackleton 2011). The diets of the three gull species are known to overlap, with all opportunistically foraging on a wide range of resources from terrestrial (natural and anthropogenic items from farmland, landfills and urban areas) and marine (intertidal prey and fish, including fishery discards) habitats (Buckley 1990; Götmark 1984; Hunt 1972; Kubetzki and Garthe 2003; Mudge and Ferns 1982). However, there is evidence of niche partitioning between the three species to reduce interspecific competition (Kim and Monaghan 2006; Ronconi et al. 2014; Steenweg et al. 2011; Washburn et al. 2013).

Our aims for this study are twofold: first, to determine the level of niche partitioning, in space and resource use, between the three sympatric gull species breeding on the same island within the same year by determining their respective foraging ranges, spatial distributions and habitat selection at the individual and population level; second, to understand how any spatial or resource niche partitioning among the three gull species may lead to differential interactions with human activities, including licensed control and other management activities, in the region, specifically in relation to offshore renewable energy developments, such as wind farms, coastal development, landfill sites, urban areas and farmland.

## 2 Methods

### 2.1 Study Site

**FIGURE 1:**
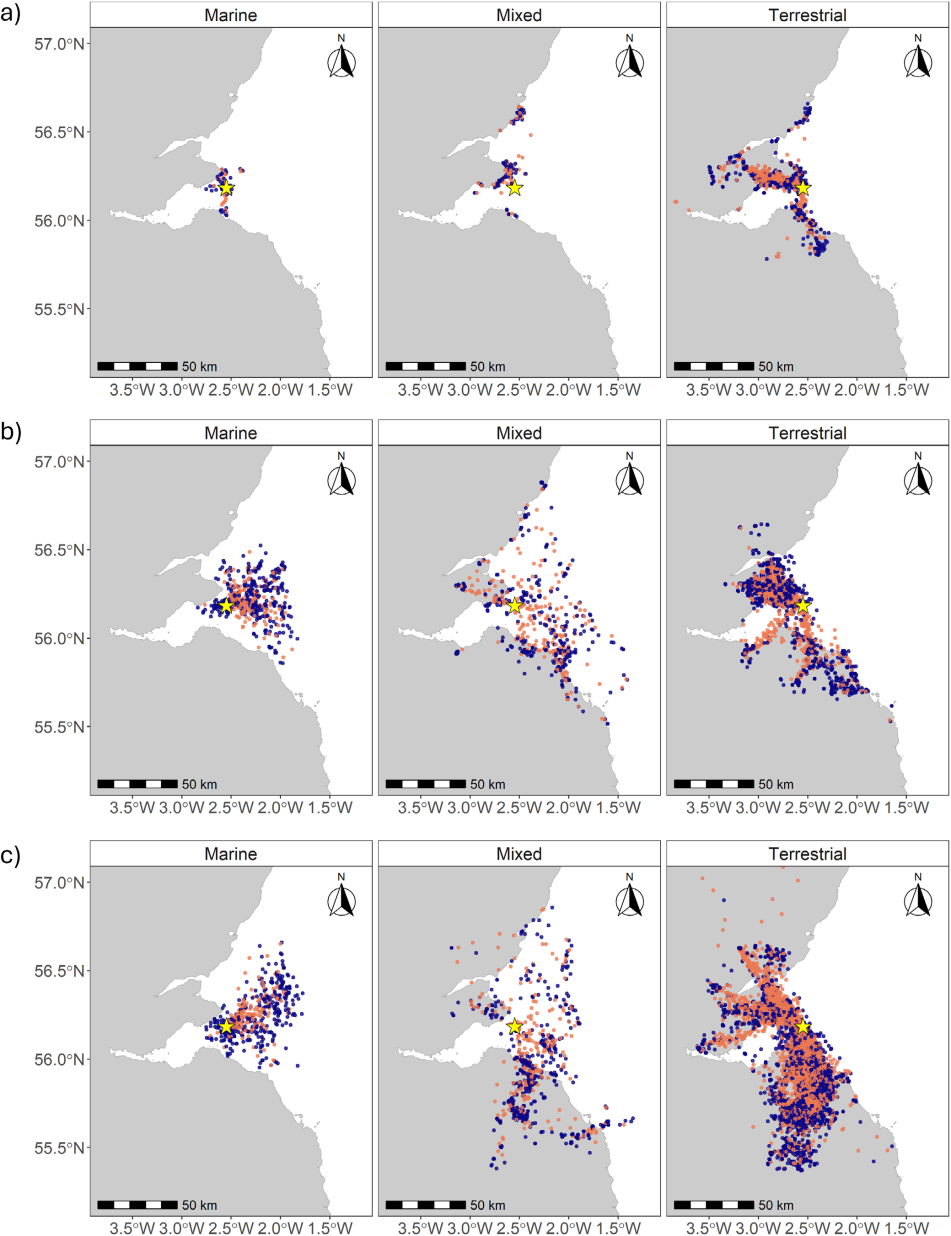
Location of fixes post‐EMbC classification during the 2021 breeding season for (a) Great Black‐backed Gulls (*n* = 10), (b) Herring Gulls (*n* = 12) and (c) Lesser Black‐backed Gulls (*n* = 31) across all years tracked classified by terrestrial, mixed and marine foraging trips. Locations identified as commuting are shown in orange and all other locations are shown in blue. The yellow star depicts the Isle of May colony location. The Scotland mainland is shown in grey and the sea in white. See Figure A1 for a map of the study region in the broader context of the UK.

**FIGURE A1:**
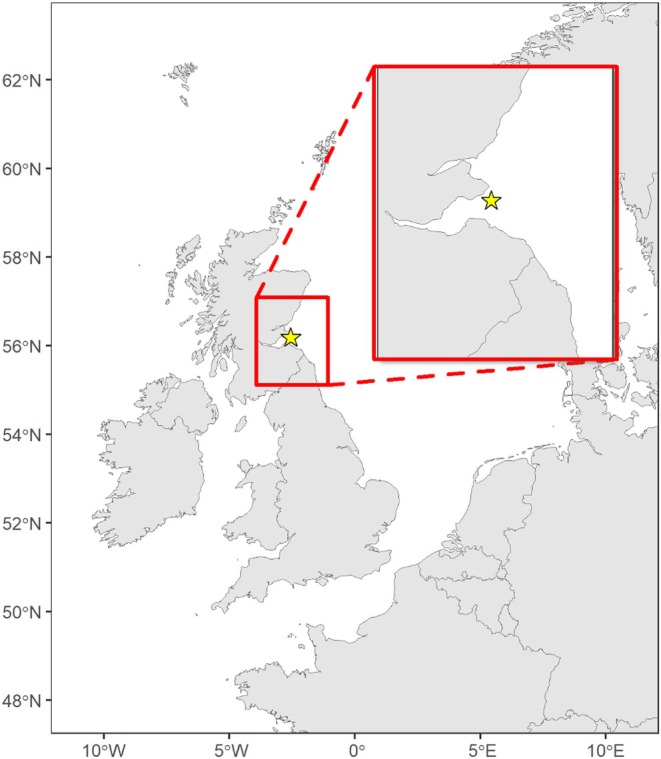
Location of the study region (within the red box) in the broader context of the UK and north‐west Europe. The yellow star depicts the Isle of May colony location.

Fieldwork was conducted on the Isle of May National Nature Reserve (56.182, −2.550; Figure 1, Figure A1) within the designated Forth Islands Special Protection Area (SPA). The Isle of May is located c. 8 km offshore within the Firth of Forth, Scotland, and is 1.5 km long and 0.5 km at its widest point. The island is also a designated Site of Special Scientific Interest (SSSI), a Site of Community Interest (SCI) and a Special Area of Conservation (SAC). Breeding population estimates for the three gull species in 2021 were 5168 Apparently Occupied Nests (AONs) for Herring Gulls; 1739 AONs for Lesser Black‐backed Gulls; and 120 AONs for Great Black‐backed Gulls (Outram and Steel 2021). The landscape surrounding the Firth of Forth is predominantly agricultural land and small villages and towns, transitioning to more urban areas near the City of Edinburgh. The coastline features a mix of intertidal habitat, sandy shores and cliffs, interspersed with coastal towns and harbours.

### 2.2 Data Collection

Adult breeding gulls were captured on the nest during mid‐late incubation or early chick‐rearing between 1 May and 4 June during the 2019 to 2021 breeding seasons (Table A1) using a wire mesh walk‐in trap (Bub 1991) or a remote‐controlled noose trap. A solar‐powered GPS device was attached to 67 individuals across species (11 Great Black‐backed Gulls, 16 Herring Gulls and 40 Lesser Black‐backed Gulls), using a thoracic cross‐strap Teflon, non‐permanent (‘weak‐link’), harness design, allowing the harness to drop off after a certain amount of time, typically up to 4 years (see Anderson et al. 2020; Clewley, Clark, et al. 2021; Langlois Lopez, Daunt, et al. 2023). This method of long‐term attachment meant that data were available for returning individuals in years after those when they were tagged, if the tag was still fitted and working (Clewley, Clark, et al. 2021). Only one member of a breeding pair was tagged in the same year. All catching, ringing and device deployment were carried out by British Trust for Ornithology ringing permit holders with relevant Special Methods Technical Panel (SMTP) licences.

In 2019, UvA‐BiTS (University of Amsterdam Bird Tracking System) GPS devices (Model 5CDLe; 13.5 g; 62 × 25 × 11 mm; Bouten et al. 2013) were deployed on 25 Lesser Black‐backed Gulls. These GPS devices remotely downloaded data to a central base‐station network placed in strategic locations on the Isle of May to cover areas where the captured Lesser Black‐backed Gulls were nesting.

In 2019 and 2021, three and 12 additional Lesser Black‐backed Gulls, respectively, were fitted with Movetech Telemetry devices (Flyway‐18; 18 g; 50 × 26.5 × 14.5 mm) which utilised the Global System for Mobile Communications (GSM) 2G network to transmit data directly to an online telemetry data repository (www.movebank.org). Herring Gulls and Great Black‐backed Gulls were only fitted with Movetech Telemetry devices. Flyway‐25 (25 g; 57.5 × 26.5 × 19 mm) devices were deployed on to two Herring Gulls in 2019 and 11 Great Black‐backed Gulls in 2021. Flyway‐18 (18 g; 50 × 26.5 × 14.5 mm) devices were deployed on to 14 Herring Gulls in 2021. The percentage mass of attachments to the birds' body mass was 2.71% ± 0.19% (Movetech) and 2.41% ± 0.23% (UvA) for Lesser Black‐backed Gulls; 2.64% ± 0.25% for Herring Gulls; and 2.00% ± 0.23% for Great Black‐backed Gulls.

UvA‐BiTS GPS devices were set to take positional fixes every 15 min when the gulls were at the colony (defined by a square geofence around the island) and every 5 min when individuals were away from the colony. When the battery was at maximum charge, away from the colony, sampling rates were increased to a fix every 10 s. For Herring and Lesser Black‐backed Gulls, Movetech devices were initially set to record one fix every 60 min between 08:00 and 20:00 and 180 min between 20:00 and 08:00 overnight (to conserve battery power). However, after 2 weeks of monitoring battery levels, these settings were remotely updated to take fixes every 30 min during the day and every 90 or 120 min during the night. The sampling interval was lengthened to 120 min to conserve battery power during the winter (October–March). For Great Black‐backed Gulls, the Movetech devices were set to record a fix every 20 min between 04:00 and 22:00 and every 180 min outside this period.

To assess device effects, we also captured additional breeding adults that were handled and ringed but not tagged (‘controls’): 47 Lesser Black‐backed Gulls (28 in 2019 and 19 in 2021); 29 Herring Gulls (19 in 2019 and 10 in 2021); and 23 Great Black‐backed Gulls (2021). All tagged and control gulls were measured (head‐bill length, gonys depth, maximum wing chord and body mass) and were fitted with a unique metal and alpha‐numeric colour‐ring to allow individual identification in the field.

### 2.3 Potential Device Effects

Due to restrictions related to COVID‐19, in 2020 and 2021, inadequate monitoring data were collected to test whether breeding success differed between tagged and control Herring or Lesser Black‐backed Gulls. During the 2019 breeding season, for Lesser Black‐backed Gulls, there was no indication that clutch size and hatching rate differed between tagged and control individuals (Table A2). Previous assessments of deploying similar devices to these two species using harnesses did not find any significant adverse impacts on return rates or productivity (Thaxter et al. 2014; Clewley, Clark, et al. 2021). Breeding success in 2021 was reduced in tagged Great Black‐backed Gulls compared to control (handled) and control (not handled) individuals, attributed to lower hatching success rates (Langlois Lopez, Daunt, et al. 2023). Specifically, only three of the 10 Great Black‐backed Gulls within this study successfully fledged chicks, with nest failure attributed to the tagging process (Langlois Lopez, Daunt, et al. 2023). Caution is therefore required when interpreting the results from this species as the majority of data were from individuals with non‐active nests, and we cannot rule out that the harness attachment altered their behaviour.

We were able to assess the return rates of tagged and control Lesser Black‐backed Gulls to the Isle of May, with no significant difference observed between the two groups and return rates in the year following tagging (Kruskal–Wallis chi‐squared test: $\chi_{2}^{2}$ = 0.010, *p* = 0.922; Table A3) or across all years up until the breeding season of 2022 (Binomial mixed‐effect model with year and individual as random effects; *β* = −0.06, $\chi_{1}^{2}$ = 0.011, *p* = 0.916). We did not compare return rates between different tag types due to insufficient power given the samples sizes (Table A1). For Herring Gulls, few individuals were tagged in 2019, with resighting efforts in 2020 prevented by COVID‐19. Resighting rates in 2022 of individuals tagged in 2021 were lower than control individuals but not significantly so (Pearson's chi‐squared test: $\chi_{1}^{2}$ = 2.24, *p* = 0.14; Table A3). Return rates were also similar between tagged and control Great Black‐backed Gulls; however, one individual died 5 days after tag deployment, potentially due to the harness attachment (see Langlois Lopez, Daunt, et al. 2023).

For Lesser Black‐backed Gulls, to check for potential long‐term effects of tagging (Kentie et al. 2024), we also tested whether the year of tagging influenced their foraging trip metrics during 2021, between June and August. We selected these months as in 2021 individuals were only tagged in late May, whilst time away from the colony increases from August once chicks have fledged (Thaxter et al. 2015). We found no significant effect of tagging year (2019 or 2021) on the maximum distance of a trip from the colony (i.e., foraging range; generalised linear mixed model with individual as a random effect: *z* = −0.013, *p* = 0.999) or trip duration (*z* = −1.760, *p* = 0.078).

### 2.4 Data Processing

**FIGURE A2:**
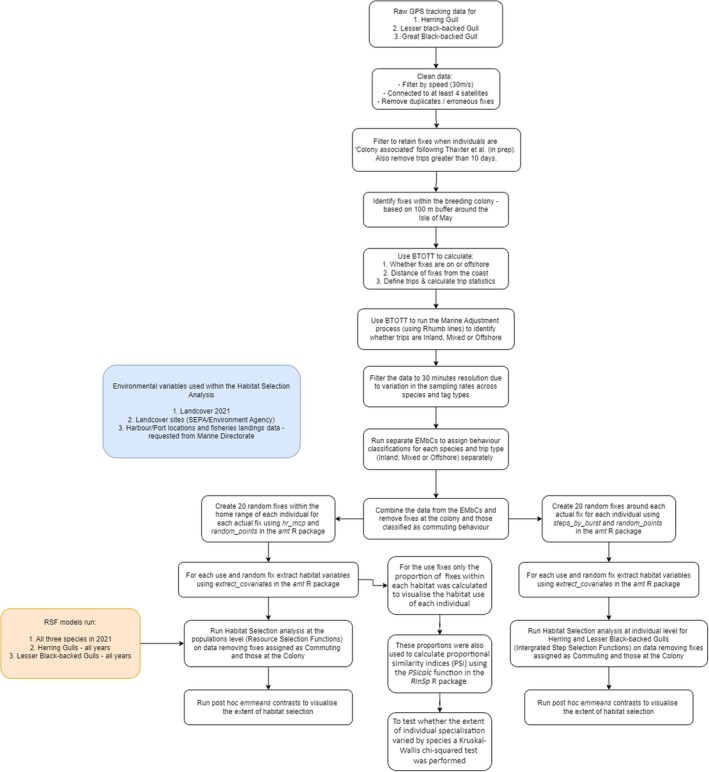
Analytical Workflow showing how the data from the GPS devices was processed and analysed.

All data processing and analyses were carried out in R version 4.3.2 (R Core Development Team 2023; see Figure A2 for our analytical workflow). Throughout, we report means and standard deviations (SD).

The raw GPS data downloaded from the deployed devices were cleaned using the *MoveRakeR* R package (Thaxter 2025) to remove inaccurate positions by excluding GPS positions obtained from three or fewer satellites and where trajectory (ground) speeds were greater than a speed threshold of 30 m/s (Shamoun‐Baranes et al. 2016; Clewley, Barber, et al. 2021). Movetech devices recorded manufacturer specific metadata (‘flt:switch’ values) on the validity of the GPS fix obtained and only ‘good’ fixes were retained for analysis. Data from one Great Black‐backed Gull and two Herring Gulls were removed from the analysis because tags had only intermittently recorded data.

As we were interested in habitat use when the gulls were associated with the Isle of May, we limited analysis to GPS fixes within a ‘colony‐associated’ period defined as from the date an individual was first fitted with a tag or first returned to the Isle of May each year to the date it left the colony (Thaxter et al., in review). We also excluded long trips, typically associated with the pre‐ and post‐breeding period, as outliers where individuals were away from the colony for more than 10 days. In addition, we removed one 3‐day trip from a Lesser Black‐backed Gull (5851) outside the study region during the breeding season (4–7 June 2021; > 250 km from the colony to the north). The colony‐associated period included ‘core‐breeding’: defined as when individuals had active nests containing eggs or chicks (i.e., covering incubation and chick‐rearing) as well as pre‐breeding and post‐fledging (Thaxter et al., in review).

### 2.5 Defining Trips

To define trips, we created a 100 m buffer around the Isle of May colony boundary using the *buffer* function in the *terra* R package (Hijmans 2022). Sequential trips for each individual were identified when an individual left and re‐entered this buffer using the *MoveRakeR* R package (Thaxter 2025). All GPS fixes outside the buffer were classified as being away from the colony on a foraging trip, whereas all fixes within the buffer were classified as being at the colony. As gaps between GPS fixes occasionally occurred due to battery levels dropping, we defined trips with > 5 h between consecutive fixes as incomplete. Only complete trips were used to calculate trip statistics.

We first classified all fixes as either terrestrial or marine, with fixes recorded in areas below the mean low water mark boundary defined as marine (GEBCO, www.gebco.net). Given that the gulls from the Isle of May have to travel over marine habitat to reach terrestrial foraging areas, we separated inland (terrestrial) trips from the mixed and offshore trips by using a bespoke technique using two rhumb lines plotted from the colony (Thaxter et al. in review). Trips that contained fixes within the rhumb lines and a distal point inland were classified as terrestrial, whereas trips that contained fixes inland and outside the rhumb lines were classified as mixed. Trips with only offshore fixes were classified as marine. Therefore, for each species, trips were classified as either terrestrial, marine or mixed (Figure 1).

### 2.6 Behavioural Classifications

For the habitat selection analysis, we wanted to focus on where the gulls were likely to be foraging or using habitats and therefore, we removed commuting fixes when travelling to and from the breeding colony. To classify the behaviour of the gulls during foraging trips, and therefore identify likely commuting fixes, we used the *Expectation‐Maximization binary Clustering* (*EMbC*) R package (Garriga and Bartumeus 2016). This analysis used an *EMbC* algorithm to assign four behaviour categories based on the speed and turning angles of successive positional fixes from tracking data: high velocity/low turning angle (HL), high velocity/high turning angle (HH), low velocity/low turning angle (LL) and low velocity/high turning angle (LH) (Garriga and Bartumeus 2016).

Due to the different sampling rates of the device types across the three gull species, especially with the 10 s data from the UvA devices, we thinned the higher‐resolution GPS data to 30 min with a tolerance threshold of 0.4 (i.e., 12 min) to ensure comparable behaviours were classified by the EMbC across species. We ran separate EMbC classifications for each of the three species (Herring, Lesser Black‐backed and Greater Black‐backed Gull) and trip type (terrestrial, offshore and mixed); resulting in nine EMbC models. For all species and trips, we assumed the high velocity/low turning angle (HL) category reflected direct flights to and from the breeding colony and therefore assigned these fixes as commuting. The remaining three behaviour categories were assumed to describe searching, foraging or resting/preening behaviours. It should be noted that some commuting activity was still likely captured within these other categories; however, this approach excluded the most obvious/direct commuting flights between the colony and foraging areas. No pre‐ or post‐smoothing was undertaken before or after running the EMbC models.

### 2.7 Environmental Covariates

To identify the habitats used by the three gull species, we used the Land Cover Map 2021 (LCM2021) 25 m raster dataset, which uses composite satellite imagery to classify land parcels into 21 land cover classes (Marston et al. 2022). These land cover classes were further grouped into five broad categories, four known to be used by foraging gulls: marine (unclassified), coastal (13—saltwater, 15–19—intertidal and saltmarsh), agricultural (3—arable, 4—improved grassland) and urban (20—urban, 21—suburban), following Clewley, Barber, et al. (2021). All remaining land cover classes were pooled as ‘Other’ (1, 2, 5–12, 14).

Given that gulls can target and forage in landfill sites (captured within the urban land cover class; Hunt 1972; Belant et al. 1998), we obtained data on the location of operational landfill sites in Scotland between 2015 and 2021 from SEPA (2022). We plotted the co‐ordinates of each landfill in QGIS 3.16.3 (QGIS Development Team 2020) and used the Google Maps Satellite Imagery base layer to manually draw polygons around the assumed wider landfill site boundaries. Landfill boundary data for England were obtained as a vector shapefile from the Environment Agency (2023). The two landfill shapefiles were merged, and we created an arbitrary 100 m buffer around each site using the *buffer* function in the *terra* R package (Hijmans 2022). The resulting shapefile was then converted to a raster using the *rasterize* function in the *terra* R package (Hijmans 2022) with the area within a landfill boundary classified as 1 and the area outside the landfill polygons as 0.

To provide information on the use of ports and harbours that the gulls may have targeted, we obtained fishery landings data from the Marine Directorate between 2019 and 2023. Specifically, we obtained landed weights (kg) per species and month where landings were from six or more vessels. Where landings were from fewer than six vessels, marine species were grouped based on their higher level International Standard Statistical Classification of Aquatic Animals and Plants (ISSCAAP) codes. To establish the use of harbour areas by the three gull species, we created an arbitrary 500 m buffer around each point location using the *buffer* function in the *terra* R package (Hijmans 2022). The resulting polygon layer was converted to a raster using the *rasterize* function in the *terra* R package (Hijmans 2022) with the area within a harbour boundary classified as 1 and the areas outside the harbour as 0. To determine whether months with higher fishery landings resulted in more visits to harbours (using the number of fixes within a 500 m buffer as a proxy), we calculated the number of fixes per species, year and month within the buffer of each harbour. We then ran separate linear models for each species with the number of fixes per month as the response variable and the fishery landings weight (kg) for each corresponding harbour and month as the explanatory variable. Not all harbours had fishery landings weight (kg) for every month. However, for all three species, we found no relationship between the number of fixes in the proximity of a harbour and landing weights (*p* > 0.030). Therefore, we did not consider the fishery landings weight data in any further analysis.

### 2.8 Statistical Analysis

**FIGURE A3:**
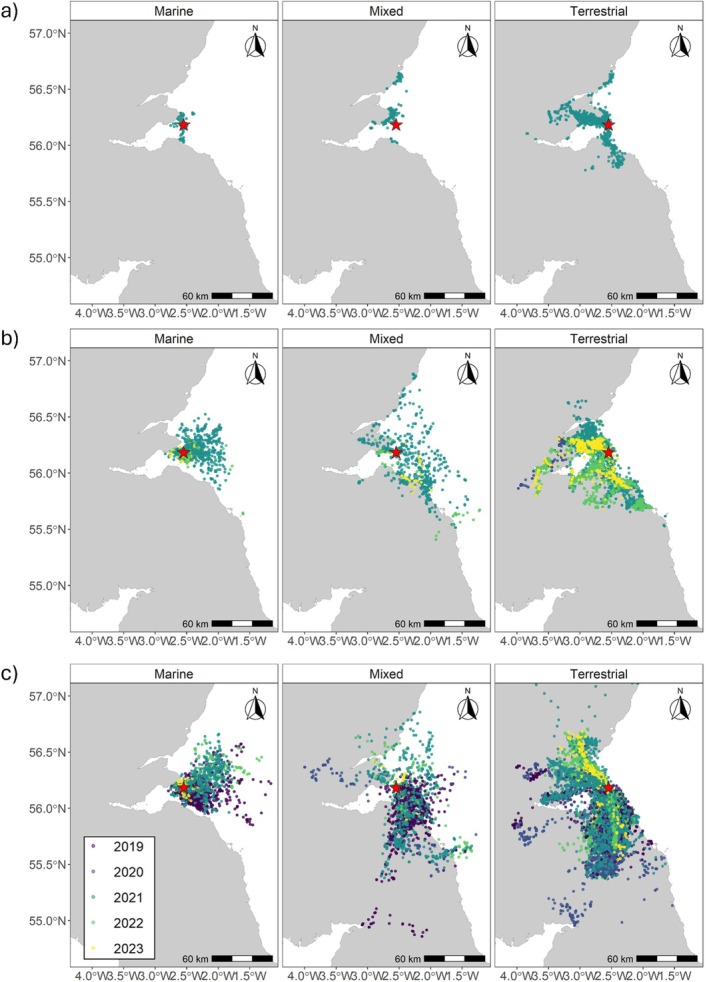
Location of fixes for (a) Great Black‐backed Gull (2021, *n* = 10) across, (b) Herring Gull (2019–2023, *n* = 14) and (c) Lesser Black‐backed Gull (2019–2023, *n* = 40), across terrestrial, mixed and marine foraging trips and for all years tracked (see legend in c) for colours.

The number of individuals and years for which tracking data were available varied among the three species: Great Black‐backed Gull (2021, *n* = 10); Herring Gull (2019–2023, *n* = 14); and Lesser Black‐backed Gull (2019–2023, *n* = 40; Table A2, Figure A3). Therefore, the analysis was focused on 2021, given that this was the only year with data available for Great Black‐backed Gulls (*n* = 10) and the year with the greatest sample size for Herring Gulls (*n* = 12, all individuals tagged in 2021) and Lesser Black‐backed Gulls (*n* = 31, 19 individuals tagged in 2019 and 12 in 2021).

To compare the spatial area used by the three gull species during 2021 when associated with the colony, we created 50% (core range) and 95% (home‐range) utilisation distributions (UD) kernels, using data with commuting fixes and those at the colony excluded. We calculated the most appropriate smoothing parameter (*h*) for each individual using a custom function in R that derives a ‘minimum’ (or adjusted) *h*‐reference bandwidth to avoid potential over‐ or under‐smoothing. This method searches iteratively for the smallest *h* over progressively smaller scales, starting with the *h*‐reference bandwidth value, and selects the smallest *h* prior to the eventual break‐up of the 95% spatial polygons. To quantify overlap between the 95% (home‐range) UDs of the three species, we calculated Bhattacharyya's affinity index values in the R package *adehabitatHR*, which range from 0 (no overlap) to 1 (identical UDs) (Bhattacharyya 1943; Fieberg and Kochanny 2005).

To test for among‐species differences in foraging ranges, calculated as the distance between the nesting site and the furthest point of a foraging trip in km, we ran a mixed‐effect model in the *glmmTMB* R package (Brooks et al. 2017) with foraging range as the response variable and species as a fixed effect. Bird ID was included as a random effect to account for variation between individuals. Post hoc contrasts were calculated using the *emmeans* R package (Lenth et al. 2020), with contrasts visualised on the response scale (km).

### 2.9 Habitat Selection at the Population Level

Resource selection functions (RSFs) were used to determine habitat selection at the home‐range scale and population level, that is, across all tracked individuals (Boyce et al. 2002; Fieberg et al. 2021; Johnson et al. 2006). For this analysis, we used the EMbC‐classified data with fixes classified as commuting and at the colony removed to form the ‘use’ locations. Individuals for which there were limited data (fewer than 100 fixes) were removed from the RSF and integrated step‐selection function (iSSF), habitat analysis (three Great Black‐backed Gulls: 1718, 1751 and 1755; three Lesser Black‐backed Gulls: 1166, 1174 and 5859). This gave us updated sample sizes of seven Great Black‐backed Gulls, 14 Herring Gulls and 37 Lesser Black‐backed Gulls across all years. For the three Great Black‐backed Gulls, the main reason for the low number of fixes, over a limited number of days available following the EMbC, was their home ranges being entirely within the Isle of May during June and July, attributed to these individuals specialising in depredating European Rabbits *Oryctolagus cuniculus* and other seabirds at the colony (Langlois Lopez 2023). Within this analysis, we focused on the gulls' habitat use when away from the colony as we were unable to differentiate between individuals foraging within the breeding colony or attending the nest or chicks. Our analyses of habitat selection and specialisation of Great Black‐backed Gulls are therefore biased to where individuals foraged outside the colony. However, these data are still useful to understand niche partitioning among the three species in the wider landscape, whilst acknowledging that this will be underestimated for Great Black‐backed Gulls.

Within the entire daily range (minimum convex polygon) of each individual, we created 20 random fixes for each use fix using the *hr_mcp* and *random_points* functions in the *amt* R package (Signer et al. 2019). For each use and random fix, we extracted the land cover class, and whether the fix overlapped with a landfill site or harbour using the *amt*::*extract_covariates* function (Signer et al. 2019). Where fixes overlapped with a landfill site or harbour, we used these habitats instead of those extracted from the land cover class to create a single habitat variable.

To run the RSFs, we performed logistic regressions in the *glmmTMB* R package (Brooks et al. 2017) with use (1) and available (0) fixes included as a binomial response variable, weighted 1 and 20, respectively (following Muff et al. 2019). Bird ID was included as a random effect to account for variation among individuals. Ideally, it would have been preferable to add Bird ID and habitat as a random slope to account for individual differences in habitat selection; however, given our categorical habitat variable, we did not have enough data for such a model to converge. To compare habitat selection between the three gull species during 2021, the only year when all three species were tracked simultaneously, we included a two‐way interaction between species and habitat as fixed effects.

To determine whether the habitat selection of Herring and Lesser Black‐backed Gulls during 2021 reflected all the years these species were tracked, we also ran separate logistic regressions at the species level. To test for annual variation in habitat selection between years we only had a large enough sample size each year for Lesser Black‐backed Gulls (Table A2). Therefore, for Lesser Black‐backed Gulls we ran a logistic regression, as above, with a two‐way interaction between year and habitat as fixed effects. For Herring Gulls, we ran a logistic regression with habitat as a fixed effect. We did initially run this model with year as well as Bird ID as a random effect; however, there was extremely small variation between years, likely due to the small sample sizes for most years.

We evaluated all logistic regression models for goodness of fit by calculating the area under the curve (AUC) for receiver operating characteristic (ROC) curves using the *pROC* R package (Robin et al. 2011). All fitted models had AUC values > 0.7 and therefore were considered acceptable (Dardis 2015). We also calculated the marginal and conditional *R* ^2^ values (Table 1).

**TABLE 1:** Resource selection function model fit from the logistic regressions of the three gull species comparison in 2021, Lesser Black‐backed Gulls and Herring Gulls assessed by the area under the curve (AUC) as well as the conditional and marginal *R* ^2^.

Logistic regression models	*R* ^2^		AUC
	Conditional	Marginal	
Three gull species (2021)	0.282	0.243	0.742
Lesser Black‐backed Gull	0.232	0.210	0.709
Herring Gull	0.342	0.315	0.708

To determine which habitats individuals had a greater selection for, and whether there were differences among species (as well as years for the Lesser Black‐backed Gulls), we ran post hoc contrasts using the *emmeans* R package (Lenth et al. 2020). For the two‐way interactions, contrasts were visualised within interaction levels on the response scale (extent of habitat selection).

### 2.10 Habitat Selection and Specialisation at the Individual Level

Given that variation in habitat use between individual gulls can be considerable, we also tested habitat selection from the perspective of the individual using iSSFs. Compared to RSFs, iSSFs provide information at the finer scale of each step between two consecutive fixes (Avgar et al. 2016). For each observed step, we generated 20 random available steps using the *amt::random_steps* function (Signer et al. 2019). Using the same habitat classifications as in the RSFs, we extracted the habitat associated with the end of each observed (use) and random available step using the *amt::extract_covariates* function (Signer et al. 2019).

An iSSF was run for all three gull species for 2021 with all commuting and colony fixes removed. We ran the conditional logistic regression model with habitat and Stratum ID as the main covariates. Stratum ID was included to pair each observed step with the 20 generated random available steps. Whether steps were observed (1) or available (0) was included as a binomial response variable. For the iSSF, agriculture was included as the reference habitat given this habitat was widely used by all individuals, and to help with the interpretation of the outputs.

**FIGURE A4:**
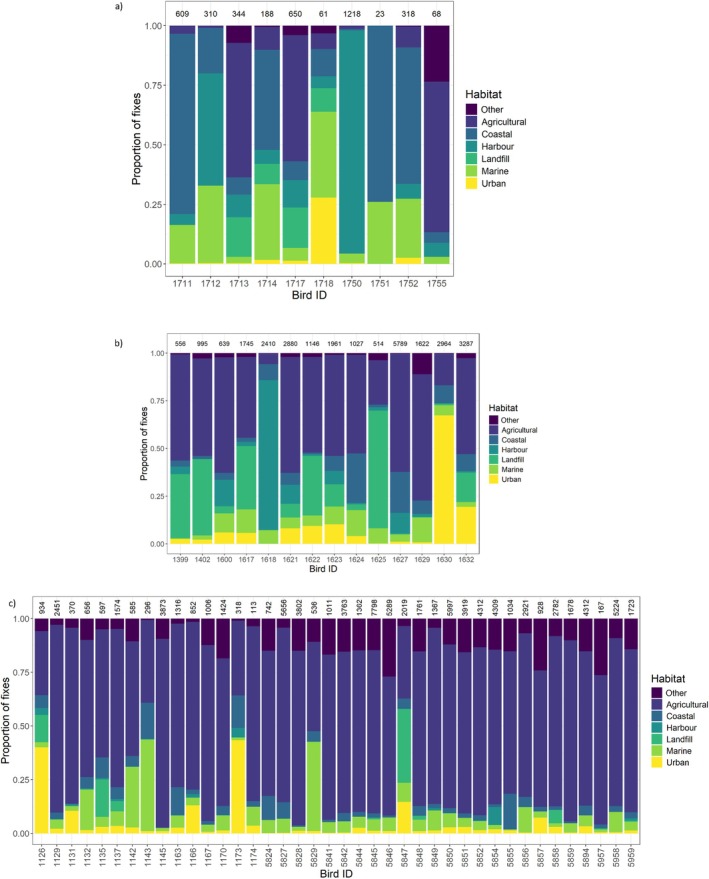
Habitat use of individual tracked (a) Great Black‐backed Gulls, (b) Herring Gulls and (c) Lesser Black‐backed Gulls during the colony‐associated period. The number above each bar refers to the number of fixes. This is based on GPS fixes following the Expectation‐Maximization binary Clustering with commuting and colony fixes removed. Data are pooled across years (2019–2023).

To investigate habitat specialisation of individual gulls, we calculated proportional similarity indices (PS_*i*_) following Bolnick et al. (2007), using the *PSicalc* function in the *RInSp* R package (Zaccarelli et al. 2013). We included 999 replicates to assess the statistical significance of the PS_*i*_ values by comparing the observed values against a null model using Monte Carlo resampling (Bolnick et al. 2002). For this analysis, we calculated the proportion of use fixes (excluding those at the colony and classified as commuting) that fell within each habitat category for each individual (Figure A4). PS_*i*_ is a measure of individual specialisation based on habitat use relative to the mean habitat use at the population level. A value of 0 indicates an absolute habitat specialist and 1 an absolute habitat generalist (Schoener 1968; Bolnick et al. 2002). For each individual, PS_*i*_ was calculated for the entire colony‐associated period for each species separately. To test whether the extent of individual specialisation varied by species, we performed a Kruskal–Wallis chi‐squared test with PS_*i*_ values as the response variable and species as the explanatory variable.

Finally, we used Pianka's niche overlap index to calculate pairwise niche overlap in habitat use between the three gull species (Liordos and Kontsiotis 2020; Pianka 1974). Using the proportion of use fixes in each habitat category (as calculated above), we calculated overlap indices using the *piankabio* function in the *pgirmess* R package (Giraudoux 2024). An overlap value of 0 indicates no niche overlap between species, whereas a value of 1 indicates complete overlap. Overlap values were categorised as low (0.00–0.39), intermediate (0.40–0.60) or high (0.61–1.00), following Grossman (1986).

## 3 Results

**FIGURE 2:**
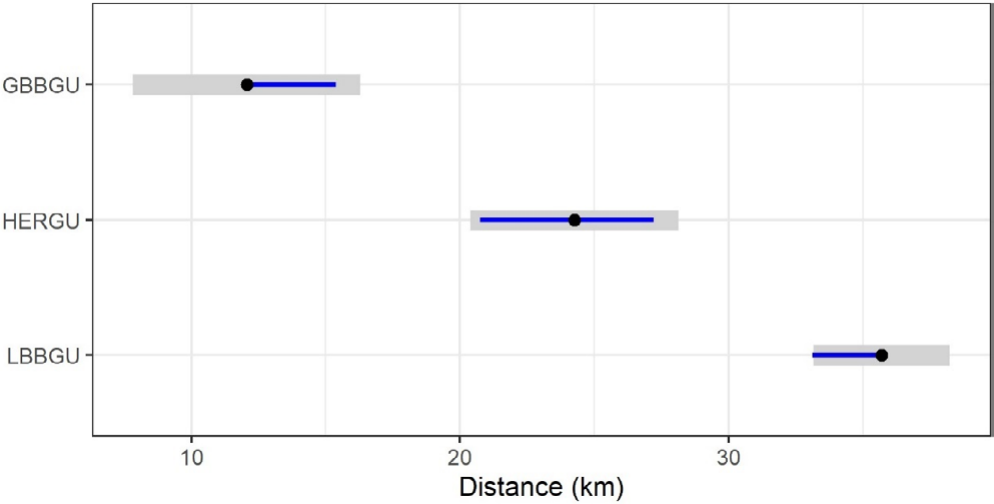
Foraging range comparisons for the three gull species tracked during the 2021 breeding season. Points show the contrast estimates, grey shading the 95% confidence intervals, and blue lines indicate pairwise comparisons; if a blue line from one species does not overlap that of another, the difference between them is significant. Based on complete trips from 10 Great Black‐backed Gulls (GBBGU: 994 trips), 12 Herring Gulls (HERGU: 1539 trips) and 31 Lesser Black‐backed Gulls (LBBGU: 2230 trips).

During 2021, when all three species were tracked simultaneously, we obtained adequate data to analyse the breeding season space use of 10 Great Black‐backed, 12 Herring and 30 Lesser Black‐backed Gulls (Table A2). When individuals were associated with the colony, most trips were to terrestrial or coastal habitats (85.0% ± 7.5% across species) to the north or south, although some individuals also made marine (6.6% ± 4.5%) or mixed (marine and terrestrial; 8.4% ± 6.3%) trips (Figure 1). This is also highlighted by the core range areas of the three species during 2021(Figure 2).

**FIGURE 3:**
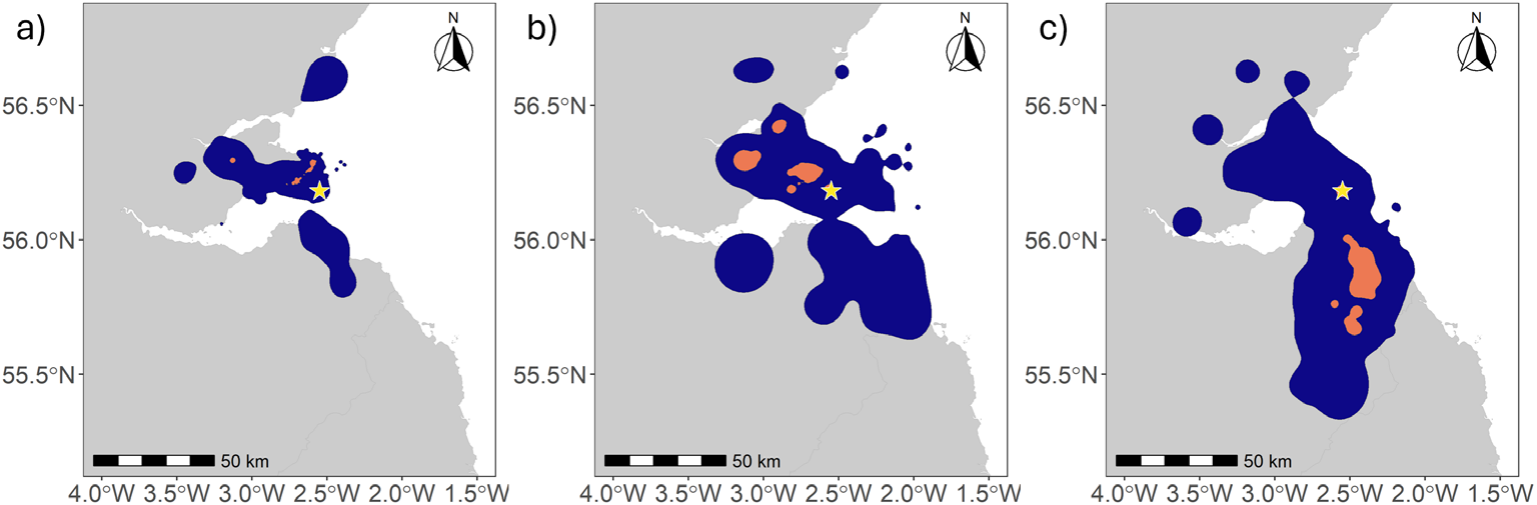
Species‐level core (50%—orange) and home (95%—blue) ranges for (a) Great Black‐backed Gulls (*n* = 10), (b) Herring Gulls (*n* = 12) and (c) Lesser Black‐backed Gulls (*n* = 31) during the 2021 breeding season. The yellow star depicts the Isle of May colony. The Scotland mainland is shown in grey and the sea in white.

During 2021 when the gulls were associated with the colony, the core and home ranges of Great Black‐backed Gulls were considerably smaller than those of Herring Gulls and Lesser Black‐backed Gulls, with limited overlap in home ranges (BA Index of 0.10 for Herring and of 0.09 for Lesser Black‐backed Gulls; Figure 3). There was also limited overlap between the home ranges of Herring and Lesser Black‐backed Gulls (BA Index = 0.11). Away from the colony, within their core ranges, both Great Black‐backed and Herring Gulls tended to target areas to the north‐west of the colony, whereas Lesser Black‐backed Gulls targeted areas to the south (Figure 3).

Comparisons of foraging ranges for the three gull species during the 2021 breeding season show the significantly greater foraging range of Lesser Black‐backed Gulls (Mean ± SD: 34.9 ± 16.5 km, Maximum: 279.0 km) than Great Black‐backed Gulls (Mean ± SD: 12.4 ± 12.0 km, Maximum: 93.4 km: Contrasts: *t* = −9.41, *p* < 0.001) and Herring Gulls (Mean ± SD: 22.2 ± 13.8 km, Maximum: 94.8 km; Contrasts: *t* = −4.85, *p* < 0.001; Figure 3). The foraging range of Herring Gulls was also significantly greater than that of Great Black‐backed Gulls (Contrasts: *t* = −4.18, *p* < 0.001; Figure 2).

### 3.1 Habitat Selection at the Species Level

#### 3.1.1 Between Species Comparisons

**FIGURE 4:**
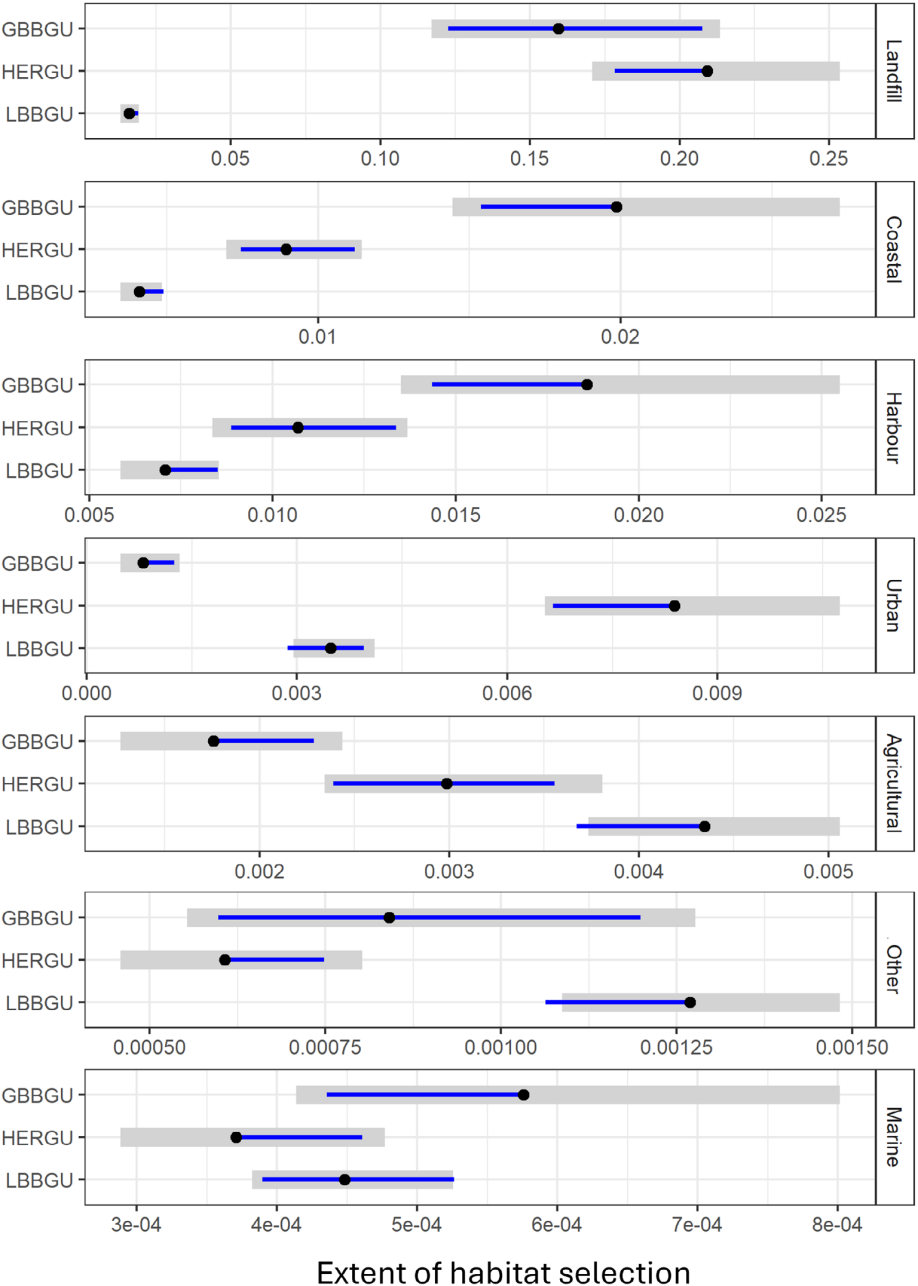
Habitat selection estimates of the three gull species, Great Black‐backed Gull (GBBGU, *n* = 7), Herring Gull (HERGU, *n* = 12) and Lesser Black‐backed Gull (LBBGU, *n* = 28), in 2021 focusing on species comparisons when colony‐associated. Post hoc estimated marginal means were extracted from the best fitting RSF logistic regression, which included a two‐way interaction between species and habitat. Due to the differing extents of habitat selection panels have different *x*‐axis scales, ordered by selection strength from the strongest (Landfill) to weakest (Marine). Points show the contrast estimates, grey shading the 95% confidence intervals, and blue lines indicate pairwise comparisons; If a blue line from one species overlaps that of another, the difference between them is not significant.

Herring and Great Black‐backed Gulls showed a stronger selection for landfill sites and coastal sites, including harbours, than Lesser Black‐backed Gulls (Figure 4). Herring Gulls selected for urban habitats to a greater extent than the other species, whilst Lesser Black‐backed Gulls showed a higher preference for agricultural habitat than Herring and Great Black‐backed Gulls. Selection for marine habitat was low relative to other habitats across all species (Figure 4).

#### 3.1.2 Within Species Comparisons

**FIGURE 5:**
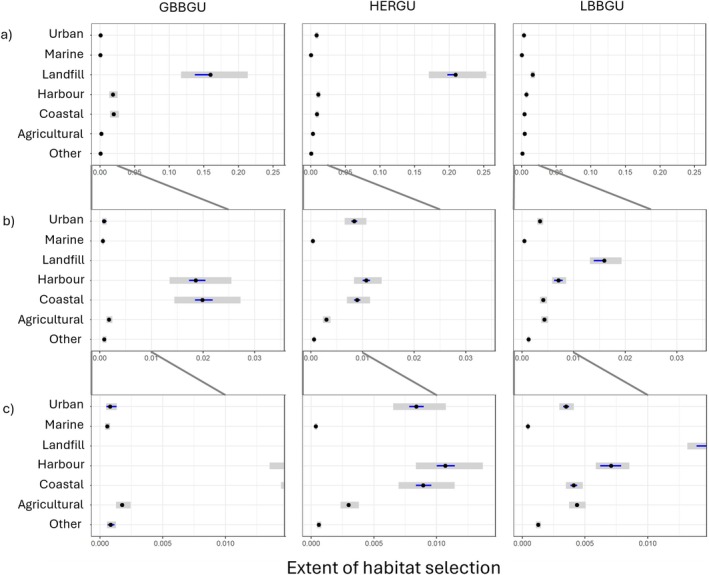
Habitat selection estimates of the three gull species, Great Black‐backed Gulls (GBBGU, *n* = 7), Herring Gulls (HERGU, *n* = 12) and Lesser Black‐backed Gulls (LBBGU, *n* = 28), in 2021 focusing on habitat comparisons within species when colony‐associated. Post hoc estimated marginal means were extracted from the best fitting RSF logistic regression, which included a two‐way interaction between species and habitat. Row (a) shows the habitat selection estimates of all habitats at full extent, without the *x*‐axis being truncated. Row (b) shows the *x*‐axis truncated to 0.035, and Row (c) shows the *x*‐axis truncated to 0.015 to better visualise habitat contrasts. Rows therefore have different *x*‐axis scales. The grey lines between plots shows the zoom linkage lines. Points show the contrast estimates, grey shading the 95% confidence intervals, and blue lines indicate pairwise comparisons; If a blue line from one species overlaps that of another, the difference between them is not significant.

**FIGURE A5:**
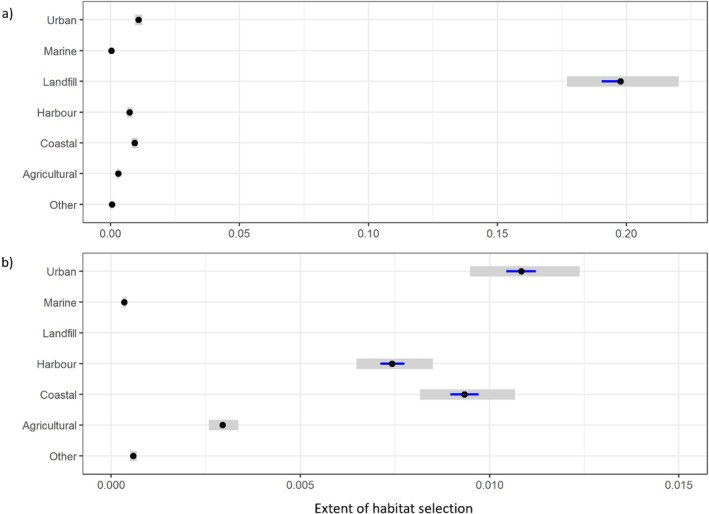
Habitat selection estimates of Herring Gulls across all years tracked (2019–2 individuals, 2020–1, 2021–12, 2022–9 and 2023–6, Total *n* = 14) when colony‐associated determined by post hoc estimated marginal means from the best fitting RSF logistic regression with habitat. Panel (a) shows the habitat selection estimates of all habitats without the *x*‐axis being truncated. Panel (b) shows the *x*‐axis truncated to better show habitat contrasts with landfill removed. Points show the contrast estimates, grey shading the 95% confidence intervals, and blue lines the significance; non‐overlap of blue lines indicates significant pairwise differences.

Focusing on habitat selection at the species level, Great Black‐backed Gulls showed the greatest selection for landfill sites, followed by coastal and harbour habitats (Figure 5). Selection estimates for the remaining habitats were low, although agricultural was selected for to a greater extent than urban and marine habitats. Herring Gulls also showed the greatest selection for landfill sites, followed by harbour, and then coastal and urban habitats during 2021 (Figure 5). Agricultural habitats were selected to a lesser extent, but more so than marine habitats. This pattern of habitat selection was similar when considering data across all 5 years Herring Gulls were tracked. However, across all 5 years, urban was the most selected for habitat after landfill for Herring Gulls, followed by coastal and harbour habitats and then agriculture (Figure A5).

**FIGURE A6:**
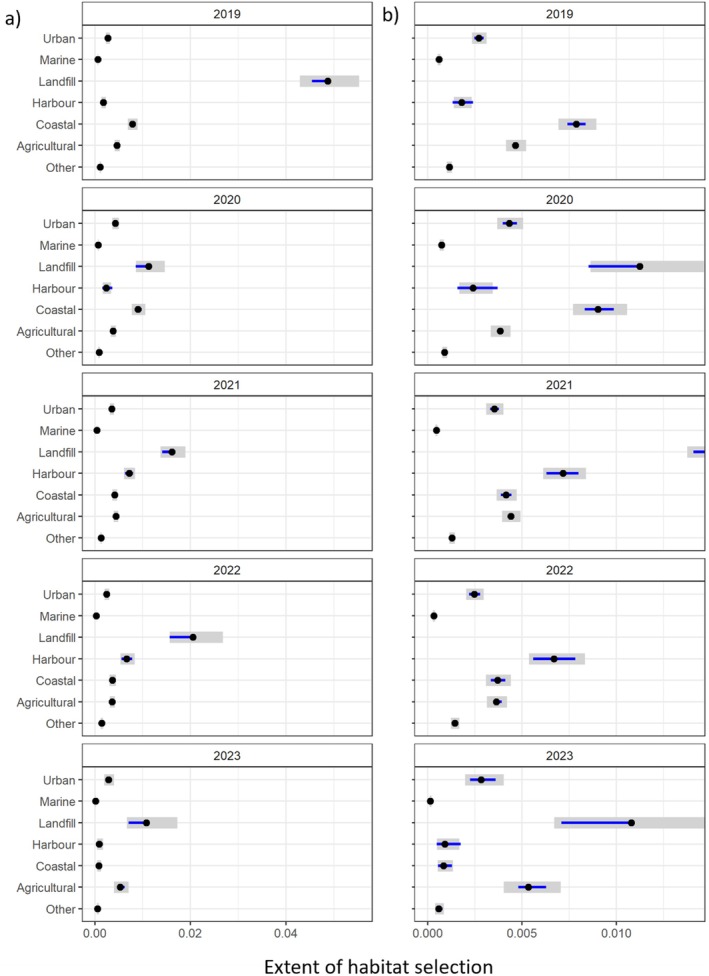
Habitat selection estimates of Lesser Black‐backed Gulls across all years tracked (2019–28 individuals, 2020–20, 2021–31, 2022–18 and 2023–6, Total = 40) when colony‐associated determined by post hoc estimated marginal means from the best fitting RSF logistic regression with a two‐way interaction between year and habitat. Panel (a) shows the habitat selection estimates of all habitats without the *x*‐axis being truncated. Panel (b) shows the *x*‐axis truncated to better show habitat contrasts. Points show the contrast estimates, grey shading the 95% confidence intervals, and blue lines the significance; non‐overlap of blue lines indicates significant pairwise differences.

Lesser Black‐backed Gulls showed the strongest selection for landfill sites, followed by harbour, and to a lesser extent coastal and agricultural habitats, then urban habitats (Figure 5). Selection for marine habitats was again very low. When considering all 5 years of data, there was variation in habitat selection among years (Figure A6). Despite this variation, landfill was the most selected habitat in most years and breeding periods, typically followed by either coastal (in 2019 and 2020) or harbour (in 2021 and 2022) habitats. There was weaker selection for agriculture and urban sites across years. Agriculture was more strongly selected for than other habitats in 2023, except landfill, but this involved a smaller sample of six individuals. In all years, marine habitat consistently had low selection estimates. It should be noted that despite the strong selection for landfill sites, only a small proportion of Lesser Black‐backed Gull use fixes (< 1%) fell within landfill sites (Figure A4). In contrast, a high number of fixes (71.5% ± 15.5%) fell within agricultural habitats; therefore, these results indicate that the Lesser Black‐backed Gulls were using this habitat largely in relation to its availability (Figure A4).

### 3.2 Habitat Selection and Specialisation at the Individual Level

**FIGURE 6:**
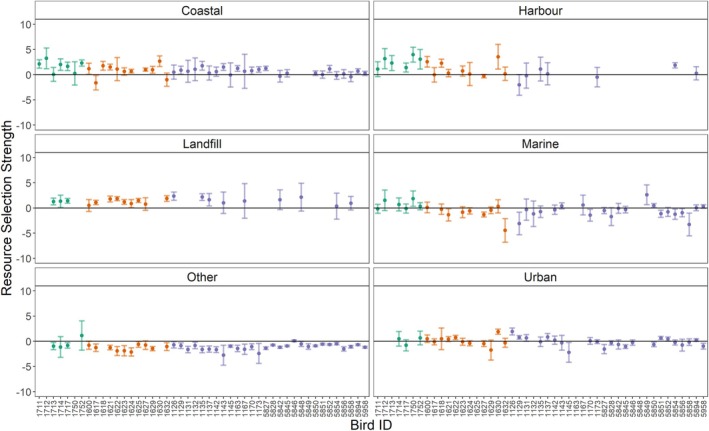
Resource selection coefficients and 95% confidence intervals for each individual Great Black‐backed Gull (green, *n* = 7), Herring Gull (orange, *n* = 12) and Lesser Black‐backed Gull (purple, *n* = 28) and habitat category included within the integrated step‐selection model (SSF) with agriculture as the reference habitat. Confidence intervals that do not overlap with zero indicate a selection (positive values) or avoidance (negative values) of that habitat relative to agriculture. Confidence intervals that overlap zero indicate no selection or avoidance. For some individuals and habitats, no coefficients were estimated due to that habitat not being used or only to a small extent.

During the 2021 breeding season, there was considerable variation in the extent to which individuals selected or avoided each habitat across species (relative to agriculture), with many individuals showing no strong selection or avoidance (Figure 6). Most Great Black‐backed Gulls showed selection for coastal habitats, including harbours, in agreement with the population‐level results. Four of seven included tracked Great Black‐backed Gulls visited landfill sites (Figure A4) and three of these showed selection for this resource. Four individuals also visited urban areas, but there was no selection or avoidance of this habitat by these individuals.

Most individual Herring Gulls showed selection for coastal habitats (seven of 11 individuals) and landfill sites (six of nine individuals), in agreement with the population‐level results (Figure 6). The result for harbours and urban areas was more mixed, indicating that these habitats were strongly selected for by a small number of individuals, which drove the population‐level selection. There was no individual selection for marine habitat, with two individuals avoiding this habitat and the remaining using this habitat in relation to its availability with the gulls' foraging range.

For 17 of the 31 Lesser Black‐backed Gulls, at least one fix overlapped with landfills, whereas for 11, at least one fix overlapped with harbour areas (Figure A4). However, for only three individuals did more than 1% of fixes occur within these two habitats. Furthermore, only three individuals showed a selection for landfill sites and one for harbour areas (Figure 6), the selection for these habitats at the population level was driven by these few individuals. More individuals selected for coastal habitats (7 of 24 individuals), although most showed no selection or avoidance of this habitat. This overall lack of selection or avoidance by individuals was also the case for urban and marine habitats; hence, why no strong selection for these habitats was observed at the population level. Part of this lack of selection for these habitats, in relation to agricultural habitats, by individual Lesser Black‐backed Gulls is likely attributed to agriculture being the most frequently used habitat by this species (Figure A4).

Across the three species, most individuals were classed as being generalists due to their relatively high PS_*i*_ values (mean specialisation index across species = 0.68, permutation *p* = 0.001) and used a range of habitats. Only one individual (Herring Gull 1618) had a PS_*i*_ value < 0.4, indicating a higher level of habitat specialisation due to largely targeting harbours (Table A4). A further two Herring Gulls had PS_*i*_ values less than 0.5, as well as two Lesser Black‐backed and three Great Black‐backed Gulls, also suggesting weak specialisation of these individuals compared to the population level. The extent of individual specialisation did also vary significantly across species ($\chi_{2}^{2}$ = 20.14, *p* < 0.001), with Great Black‐backed Gulls showing the greatest extent of specialisation (PS_*i*_ value of 0.52 ± 0.10, *n* = 7) and Lesser Black‐backed Gulls being the most generalist (0.83 ± 0.14. *n* = 28), with Herring Gulls being intermediate between the two (0.66 ± 0.17, *n* = 12).

In terms of niche overlap, habitat use overlap was high between Herring and Lesser Black‐backed Gulls (Pianka index value = 0.92), and between Herring and Great Black‐backed Gulls (0.66). In comparison, habitat overlap between Lesser Black‐backed and Great Black‐backed Gulls was intermediate (0.49).

## 4 Discussion

To partition resources and reduce inter‐ and intraspecific competition, individuals can differ in their habitat use and/or have spatially or temporally segregated foraging areas (Corman et al. 2016; Shlepr et al. 2021; Washburn et al. 2013). This study revealed considerable overlap in the habitat use of the three sympatric gull species when foraging away from the colony during the colony‐associated period; particularly between Herring and Great Black‐backed Gulls and Herring and Lesser Black‐backed Gulls. However, differences in habitat selection and spatial distribution of home and core areas suggest a level of niche separation. Lesser Black‐backed Gulls predominantly used terrestrial areas to the south of the colony, targeting agricultural habitat. Herring Gulls used coastal and terrestrial areas to the north, closer to the colony, and targeted coastal (intertidal) habitat and harbours, as well as urban areas and landfill sites. Similarly, Great Black‐backed Gulls also used coastal and terrestrial areas to the north but stayed closer to the colony, targeting coastal areas, with some individuals also targeting harbours and landfill sites, or predominantly foraging at the colony.

### 4.1 Spatial Distributions and Foraging Ranges

Although the home ranges of the three gull species showed some overlap when associated with the colony, their core ranges showed striking differences. The core range of Lesser Black‐backed Gulls did not overlap with those of Great Black‐backed or Herring Gulls. Great Black‐backed Gulls had a very small core range, which incorporated specific coastal and harbour areas and a landfill site. Herring Gulls also targeted similar locations; however, they made greater use of the wider landscape which encompassed these sites. This resulted in differences in the foraging ranges of the three species. Lesser Black‐backed Gulls had significantly greater foraging ranges compared to Herring and Great Black‐backed Gulls. This indicated some spatial segregation between the three species, especially with the smaller, arguably less competitive Lesser Black‐backed Gulls, which travel greater distances to obtain food (Verbeek 1977; Greig et al. 1985). Conversely, the larger Great Black‐backed Gulls could likely defend foraging territories closer to or within the colony, given their greater competitive advantage. Given the overlap in resource niches between the three species, this spatial segregation may be a mechanism to reduce interspecific competition driven by competitive exclusion and spatial avoidance (Bonnet‐Lebrun et al. 2024; Ronconi and Burger 2011).

Previous studies have shown that Lesser Black‐backed Gulls have larger foraging ranges than Herring Gulls (Thaxter et al. 2012; Woodward et al. 2024). However, few studies have reported the foraging ranges of Great Black‐backed Gulls (Woodward et al. 2024). The Great Black‐backed Gull foraging ranges observed in this study were similar, if slightly smaller, to those in Canada (Maynard 2018; Maynard and Ronconi 2018). The small foraging ranges in this study were in part influenced by four individuals that in June and July had home ranges entirely within the colony boundary where they specialised in depredating other seabirds, particularly Atlantic Puffins *Fratercula arctica*, and European Rabbits (Langlois Lopez, Clewley, et al. 2023).

### 4.2 Habitat Selection

When associated with the colony, Herring and Great Black‐backed Gulls were similar in their overall strong selection for landfill and coastal habitats, including harbours and ports. Coastal, specifically intertidal, areas are a traditional key habitat for Herring Gulls, especially at low tide (Pierotti and Annett 1991; Hüppop and Hüppop 1999), which were also important within this study. Harbour towns along nearby coastlines provide scavenging opportunities associated with tourist activities and fish landings (Beasley 2017; Foster et al. 2017). Lesser Black‐backed Gulls showed weaker selection for landfill and coastal habitats. Agricultural areas (including pastures) were the main habitat used by Lesser Black‐backed Gulls, but with weak selection for this habitat due to it being widely available within the foraging range of the colony and therefore individuals used it in relation to its relative availability. The particularly high habitat selection estimates for landfill sites across species were attributed to this habitat being rare (in terms of km^2^ coverage) in the wider environment compared to the other included habitats.

Herring Gulls showed the greatest selection for urban areas, although this was driven by a small number of individuals, as has been observed elsewhere (Lato et al. 2021). Schools, green spaces, such as parks, and shopping centres, in both coastal and inland urban areas, can provide predictable food sources for gulls (Spelt et al. 2019). For Great Black‐backed and, particularly, Lesser Black‐backed Gulls, the population‐level selection for landfill, urban and harbour habitats, was also driven by a subset of individuals. Our results highlight the importance of analysing habitat selection at both the population and individual level to understand whether population‐level patterns are representative of all individuals or driven by a subset; as well as considering absolute habitat use in addition to habitat selection.

Foraging at sea, for fish or scavenging fishery discards, can be important for Lesser Black‐backed Gulls in some regions (Isaksson et al. 2016; Kubetzki and Garthe 2003; Tyson et al. 2015), and for Great Black‐backed Gulls (Maynard et al. 2021; Washburn et al. 2013). In this study, marine areas away from the coast were not extensively used by any species, although some individuals did use this habitat, likely scavenging from fishing vessels rather than actively capturing fish (Furness et al. 1992; Camphuysen 1995). Reduced fishery discards, following EU policy changes, may have decreased marine foraging opportunities, influencing the habitat selection observed in this study (Bicknell et al. 2013; Foster et al. 2017; Sherley and Votier 2019). We also found that the amount of time gulls spent visiting harbours was not related to fishery landings weights. This may be attributed to gulls visiting harbours not only for foraging opportunities associated with landings but also to scavenge food associated with these locations being popular tourist hotspots. Previous studies have also shown limited use of marine habitats by Lesser Black‐backed Gulls at certain colonies, especially away from the coast, where individuals instead forage more in terrestrial areas, particularly agricultural habitats as we found (Coulson and Coulson 2008; Gyimesi et al. 2016; Langley et al. 2021; Spelt et al. 2019; Thaxter et al. 2015). Although food from agricultural habitats may not necessarily be the most profitable in terms of energetic and nutritional quality, this habitat is widely available and low risk (in regard to levels of competition) in comparison with foraging at sea or in urban areas, and is relatively predictable (O'Hanlon et al. 2017; van Donk et al. 2017).

Most habitats selected by the gulls in this study were associated with human activities (landfill sites, harbours, urban areas and agriculture), which may lead to interactions with humans, and potential conflict. Requests for licenced control of gulls does occur at landfill sites within the region, as well as associated with urban and agricultural habitats under risk to public health or safety and prevention of serious damage to crops and livestock (NatureScot 2020a, 2020b). Understanding how sympatric gull species use these habitats is therefore important to determine how such licenced activities may differentially impact species, and individuals, and therefore the gulls' population dynamics. For example, within this study region, Herring and Great Black‐backed Gulls will be impacted to a greater extent by licenced lethal control occurring at landfill sites, whereas in agricultural habitats, any licenced control will impact Herring and Lesser Black‐backed Gulls to a greater extent. Given the importance of these habitats to the gulls tracked within this study, licenced control has the potential to negatively impact protected populations of breeding gulls (in this case those from the Forth Islands SPA and the connected Outer Firth of Forth and St Andrews Bay Complex SPA).

Within this study, we focused on foraging ecology away from the breeding colony to determine habitat selection and interactions with human activities across the region. By excluding fixes at the colony, where individuals can obtain food through kleptoparasiting or predation of other seabirds (Busniuk et al. 2020; Källander 2006; Stenhouse and Montevecchi 1999), we likely underestimated habitat selection at the population level. This is particularly true for Great Black‐backed Gulls given that three individuals spent a large proportion of the breeding season foraging exclusively within the colony (Langlois Lopez 2023). We initially included data from these three Great Black‐backed Gulls in the habitat selection analysis; however, the results were similar to when they were excluded given the low number of fixes over a small number of days for each individual away from the colony. It is also important to note here the relatively small sample size of Great Black‐backed Gulls, as well as this data largely being from non‐breeding individuals due to nest failure attributed to the tagging process (Langlois Lopez, Daunt, et al. 2023). As stated in the methods, caution is therefore required when interpreting these results, as we cannot rule out that the harness attachment altered the behaviour of the Great Black‐backed Gulls. However, several studies have shown that habitat use and colony attendance can be similar between failed and successful gulls during the breeding season; therefore, the data are still valuable (Baert et al. 2021; Maynard et al. 2022). We found no evidence of device effects on Herring or Lesser Black‐backed Gulls; however, it is important that every tracking study checks for such effects given that they may be location specific.

Although most of our analysis focused on a single year (2021), we did have additional data across 5 years for Herring and Lesser Black‐backed Gulls. Sample sizes per year were only adequate to look at annual variation in habitat selection for Lesser Black‐backed Gulls. This showed that habitat selection was similar between 2019 and 2020, and between 2021 and 2022, which largely involved two different cohorts, the first tagged in 2019 and the second in 2021. Therefore, the differences observed between years were likely driven by variation in individual habitat preferences, although we cannot rule out changes in resource availability. For example, the weaker selection for harbours in 2020 compared to 2021 and 2022 may be attributed to COVID‐19 restrictions reducing foraging opportunities associated with tourists. Selecting the only year in which data were available for all three species removed the influence of potential inter‐annual variability on our interpretation of niche partitioning, for example, variability driven by differences in food availability and foraging areas among years (Fox et al. 1990; Mendes et al. 2018). However, it means that our understanding of how these changes may affect habitat use, and therefore the extent of niche partitioning, is likely less comprehensive. This may be further influenced by the relatively small number of individuals that were tracked during this study, particularly for Herring and Great Black‐backed Gulls, if we did not capture the full habitat use of each species at this colony. Although, at the broad scale the tracked gulls did use all habitat types that we would expect them to (Clewley, Clark, et al. 2021; Clewley, Barber, et al. 2021; O'Hanlon et al. 2022).

### 4.3 Habitat Specialisation and Niche Partitioning

Previous diet and tracking studies have found strong resource specialisation of individuals within some gull populations (Davis 1975; Juvaste et al. 2017; Maynard and Ronconi 2018; McCleary and Sibly 1986; van den Bosch et al. 2019). In this study, most individuals used a variety of habitats; however, Great Black‐backed Gulls showed the greatest specialisation and Lesser Black‐backed Gulls the least. This finding reflects their known habitat flexibility, with Herring and Lesser Black‐backed Gulls being more flexible in their habitat use than Great Black‐backed Gulls that prefer prey at higher trophic levels (Garthe and Hüppop 2004; Götmark 1984). The lack of individual specialisation in Lesser Black‐backed Gulls was driven by their predominant use of widely available agricultural habitats. In comparison, no single habitat dominated Herring or Great Black‐backed Gulls habitat use. That the three gull species did not specialise on different resources was also reflected in the limited evidence for resource niche partitioning between them.

Given that greater resource diversity typically leads to greater specialisation (Araújo et al. 2011), we might have expected greater individual specialisation and niche partitioning across the three gull species given the diversity of habitat within the vicinity of the colony, especially as specialising on specific resources can have fitness consequences associated with reduced foraging costs (Masello et al. 2013; Terraube et al. 2014; van den Bosch et al. 2019). Intraspecific competition for resources can also increase individual specialisation, although this depends on individual habitat preferences (Araújo et al. 2011; Svanbäck and Bolnick 2005). Conversely, where strong interspecific competition occurs among sympatric species, within‐species individual specialisation can be weaker (Araújo et al. 2011). Therefore, the limited specialisation observed in this study may have been due to high levels of interspecific competition for available resources. For example, reduced foraging opportunities associated with declining fishery discards at sea and at harbours (Bicknell et al. 2013; Foster et al. 2017) may have increased interspecific competition in coastal and terrestrial habitats, resulting in reduced opportunities for niche partitioning within this region. Alternatively, specialisation within species and resource partitioning between species may have been limited due to the diverse habitats across the region providing adequate, profitable foraging opportunities, resulting in low intra‐ and interspecific competition for resources within the region during the study period.

The greater specialisation observed in Great Black‐backed Gulls compared to the other two species is likely due to their greater territorial and competitive abilities (Rome and Ellis 2004), allowing them to secure preferred resources at a higher trophic level, closer to the colony. A previous UK study also found that although most Great Black‐backed Gulls were classified as dietary generalists, a small number of individuals were identified as bird or mammal specialists (Westerberg et al. 2019). Although not examined in this study, dietary partitioning is also likely a factor, as Great Black‐backed Gulls can handle and swallow whole larger prey than Herring and Lesser Black‐backed Gulls (Steenweg et al. 2011; Ronconi et al. 2014). By only focusing on habitat use away from the colony, we underestimated the extent of specialisation in Great Black‐backed Gulls at the species level as we excluded three individuals that specialised on foraging within the colony (Langlois Lopez 2023). Consequently, we also underestimated the extent of niche partitioning between Great Black‐backed Gulls and the two smaller species given the former's ability to outcompete Herring and Lesser Black‐backed Gulls for prey within the colony. This competitive advantage at the colony may reduce interspecific competition in the wider landscape, given that fewer Great Black‐backed Gulls foraged away from the colony. Despite the limitation of this study in not including the resource use of Great Black‐backed Gulls at the colony (which is the focus of a complementary study; Langlois Lopez 2023), it still provides important information on the extent of habitat selection and niche partitioning of the three gull species when away from the colony and where they are most likely to interact with human activities. That specialisation was not higher for Great Black‐backed Gulls away from the colony may indicate that no single resource met all their requirements or that resources were not predictable enough to support specialisation (MacArthur and Pianka 1966).

### 4.4 Impact on Habitat Use of Further Anthropogenic Habitat Change

To meet carbon emission targets associated with mitigating human‐induced climate change, many countries are investing in renewable energy, particularly offshore windfarms (Kumar et al. 2016; Scottish Government 2020). However, these developments can negatively impact seabirds through collision, displacement, barrier effects and habitat loss (Drewitt and Langston 2006), with gulls at high risk to mortality through collisions with turbines (Furness et al. 2013). It is therefore important to identify whether offshore windfarms negatively impact populations of protected seabirds, such as gulls breeding within SPAs. This study found limited use of the marine environment by all three gull species. However, it is important to note that we only tracked a small proportion of individuals, during the breeding season. At the time of this study, several proposed windfarms off the Firth of Forth were not yet built or operational. Gulls can be attracted to roosting on structures at sea, such as those associated with wind turbines (Cook et al. 2018; Vanermen et al. 2020), as well as to potentially increased food ability due to structures acting as artificial reefs and bans on fishing activities (Dierschke et al. 2016). Therefore, once the proposed windfarms are constructed there may be greater attraction to these areas by gulls, potentially increasing their risk of collision, highlighting the importance of obtaining post‐construction tracking data (Vanermen et al. 2015; Johnston et al. 2022). Given the extensive use of the onshore environment, particularly by Herring and Lesser Black‐backed Gulls, it is also important to consider the impact of onshore renewable developments and associated infrastructure, such as power lines on these species (Gauld et al. 2022).

Future coastal developments associated with the proposed increase in offshore windfarms are also predicted, particularly floating developments (Scottish Government 2020). These may involve considerable development at specific harbours, including infrastructure, such as pipelines and wet storage. Given the importance of coastal habitat, particularly to Herring and Great Black‐backed Gulls within this study, coastal developments will likely affect the availability of foraging, and roosting, opportunities for gulls as well as other coastal species, such as waders and waterfowl (Dugan et al. 2008; Graells et al. 2022), and result in the displacement of gulls to other habitats. Displacement from landfill sites due to closures or deterrents put in place to deter scavengers may also force individual gulls exploiting this resource to alternative habitats (Cook et al. 2008; Langley et al. 2021). Such displacement may have consequent impacts on the gulls' demographic rates, including breeding success and survival, depending on the energetic and nutritional quality of alternative available habitats within their foraging range (Pierotti and Annett 1991; Belant et al. 1998; van Donk et al. 2017; Delgado et al. 2023), as well as new conflicts arising in the habitats gulls switch to (Langley et al. 2021).

Although selection for agricultural habitat was generally weak, it was still important for Lesser Black‐backed Gulls and also Herring Gulls, as has been shown elsewhere (Coulson and Coulson 2008; Gyimesi et al. 2016; Pennycott et al. 2020). Unfortunately, we did not have high enough resolution habitat data to identify the specific resources or food that gulls were targeting within agricultural areas. However, they were likely targeting invertebrates and small mammal prey, especially associated with ploughing and mowing, as well as potentially supplementary livestock food (Camphuysen 2013). The latter may cause conflict with farmers as it takes away this food from its target, as well as concerns of disease transmission (Butterfield et al. 1983; Navarro et al. 2019; Hill et al. 2022). Given the availability of agricultural land and that it is a relatively low risk, accessible resource for gulls to exploit compared, for example, to landfill and urban areas (van Donk et al. 2019), there is a concern that displacement of gulls from preferred habitats, such as harbours and landfill sites, due to development and disturbance/closures, respectively, may lead to more gulls foraging in agricultural habitats (Langley et al. 2021). This may result in increased conflict with human interests, as well as potentially increasing competition for this resource between Lesser Black‐backed and Herring Gulls. Furthermore, switching to alternative habitats may impact the gulls' demographic rates, for example, foraging on agricultural resources may result in reduced breeding success compared to foraging on intertidal, marine or urban resources (Pierotti and Annett 1991; O'Hanlon et al. 2017).

## 5 Conclusion

This study highlights the complex foraging ecologies of three sympatric gull species, when foraging away from the colony, during the colony‐associated period. Resource partitioning was relatively low among the three species. However, differences were observed in habitat preferences, extent of specialisation and their spatial distributions, indicating some level of niche separation, which likely reduced interspecific competition. Resources associated with human activities were targeted indicating that interactions with humans may occur in urban, coastal and agricultural areas across the region. Depending on future development in the region, conflict in certain habitats, specifically agricultural, may increase. Given the overlap in resource niches, all three gull species are likely vulnerable to resource degradation or loss of access to the studied habitats, though specific changes may affect each species differently. For example, Herring and Great Black‐backed Gulls would be most impacted by the loss of access to landfill sites, whereas Herring Gulls would be particularly affected by reduced foraging opportunities in urban areas. The extent of the impact of anthropogenic habitat changes on each species would also depend on the location of these changes, given the spatial segregation of their core ranges. Although we only focused on the colony‐associated period, it is likely these changes will impact Herring and Great Black‐backed Gulls year‐round given that many individuals will be resident within the region, unlike Lesser Black‐backed Gulls which will typically migrate away from the region following the breeding season (Spina et al. 2022).

In generalist species, resource selection and specialisation are strongly influenced by trade‐offs between resource availability and predictability, as well the specific requirements of an individual at a given time of the annual cycle (Baert et al. 2021; Ceia and Ramos 2015; Masello et al. 2013; van Donk et al. 2017). Consequently, if the availability of resources changes within the region, the extent of individual specialisation and niche partitioning among species will also likely change (Araújo et al. 2011). Such shifts could, in turn, affect the vulnerability of each species to changes in resource availability with subsequent consequences on their demographic rates, for example, through reduced quality of resources or increase mortality through conflict.

By tracking individuals from several generalist species and exploring habitat selection at both the population and individual level, we can better understand how these species utilise areas of human activity and, importantly, whether all or a subset of a population may be impacted by future habitat change, which is particularly important to consider when such populations are protected. This holistic approach to understanding the foraging ecologies of sympatric generalist species can provide vital information to inform the management and conservation of gull populations and to mitigate against potential conflicts arising from habitat changes.

## Author Contributions

**Nina J. O'Hanlon:** conceptualization (equal), formal analysis (lead), investigation (equal), methodology (equal), visualization (lead), writing – original draft (lead), writing – review and editing (equal). **Gary D. Clewley:** conceptualization (equal), data curation (equal), investigation (equal), methodology (equal), writing – review and editing (equal). **Daniel T. Johnston:** conceptualization (equal), investigation (equal), methodology (equal), writing – review and editing (equal). **Chris B. Thaxter:** conceptualization (equal), data curation (equal), formal analysis (equal), methodology (equal), writing – review and editing (equal). **Samuel Langlois Lopez:** conceptualization (equal), data curation (equal), investigation (equal), methodology (equal), writing – review and editing (equal). **Lucy R. Quinn:** conceptualization (equal), funding acquisition (equal), writing – review and editing (equal). **Philipp H. Boersch‐Supan:** formal analysis (equal), writing – review and editing (equal). **Elizabeth A. Masden:** conceptualization (equal), funding acquisition (supporting), writing – review and editing (equal). **Francis Daunt:** conceptualization (equal), writing – review and editing (equal). **Jared Wilson:** conceptualization (supporting), funding acquisition (equal), writing – review and editing (equal). **Niall H. K. Burton:** conceptualization (equal), funding acquisition (equal), project administration (equal), writing – review and editing (equal). **Elizabeth M. Humphreys:** conceptualization (equal), funding acquisition (equal), project administration (equal), writing – review and editing (equal).

## Ethics Statement

Ethics approval for this study was issued by the British Trust for Ornithology's independent Special Methods Technical Panel under the UK Ringing Scheme (licence numbers: 4255, 4260 and 11,747).

## Conflicts of Interest

The authors declare no conflicts of interest.

## Data Availability

Datasets originating from the Isle of May generated and analysed during the current study are available via the movebank.org repository. Great Black‐backed Gull—https://www.movebank.org/cms/webapp?gwt_fragment=page=studies,path=study1512405681. Herring Gull—https://www.movebank.org/cms/webapp?gwt_fragment=page=studies,path=study834346398. Lesser Black‐backed Gull—https://www.movebank.org/cms/webapp?gwt_fragment=page=studies,path=study834344857. The R code that supports the findings of this study are available on Zenodo at 10.5281/zenodo.14534126.

## Appendix A

**TABLE A1:** Number of individuals captured per species on the Isle of May in 2019 and 2021, that were either fitted with GPS devices ‘tagged’, or simply fitted with a colour ring as ‘control’ birds. Number of total individuals with adequate data from those tagged in parenthesis. Great Black‐backed Gull (GBBGU), Herring Gull (HERGU) and Lesser Black‐backed Gull (LBBGU).

Year	GBBGU		LBBGU		HERGU	
	Tagged	Control	Tagged	Control	Tagged	Control
2019	0	0	25^b^ + 3^a^	28	2^a^	19
2021	11^1^	23	12^a^	19	14^a^	10
Total	11 (10)	23	40 (40)	47	16 (14)	29

^a^ Movetech Telemetry.

^b^ University of Amsterdam.

**TABLE A2:** Summary of productivity monitoring data collected from GPS tagged and untagged control Lesser Black‐backed Gulls breeding on the Isle of May in 2019. All values are mean ± Standard deviation.

Colony	Year marked	Group	Clutch size (*n*)	Proportion of eggs hatched
Isle of May	2019	Tagged	2.82 ± 0.38 (28)	0.56
		Control	2.76 ± 0.51 (28)	0.56

**TABLE A3:** Summary of return rates of GPS tagged and untagged control Lesser Black‐backed Gulls (LBBGU) and Herring Gulls (HERGU) breeding on the Isle of May between 2019 and 2022.

Species	Year tagged	Group	Number	2020	2021	2022
				Proportion re‐sighted	Proportion re‐sighted	Proportion re‐sighted
LBBGU	2019	Tagged	28	0.53	0.42	0.48
		Control	28	0.50	0.46	0.43
LBBGU	2021	Tagged	12	—	—	0.42
		Control	19	—	—	0.53
HERGU	2021	Tagged	14	—	—	0.50
		Control	10	—	—	0.80

**TABLE A4:** Summary of the GPS data collected from the three gull species, Great Black‐backed Gull, Herring Gull and Lesser Black‐backed Gull, captured on the Isle of May between 2019 and 2023, when associated with the colony.

Species	Bird ID	Year tagged	Year	First date associated with the colony	Last date associated with the colony	No. of days	No. of fixes (excluding those at the colony and when commuting)	PS_*i*_ in 2021	VarPS_*i*_ in 2021
Great Black‐backed Gull	1711	2021	2021	19‐05‐2021 06:14	28‐08‐2021 12:30	53	609	0.203779	0.000126
Great Black‐backed Gull	1712	2021	2021	14‐05‐2021 08:42	27‐08‐2021 11:34	52	310	0.20197	4.43E−05
Great Black‐backed Gull	1713	2021	2021	17‐05‐2021 07:18	08‐08‐2021 15:50	55	344	0.828392	0.00065
Great Black‐backed Gull	1714	2021	2021	16‐05‐2021 08:12	27‐08‐2021 11:43	37	188	0.334718	0.000773
Great Black‐backed Gull	1717	2021	2021	23‐05‐2021 06:25	25‐08‐2021 16:52	75	650	0.798079	0.000359
Great Black‐backed Gull	1718	2021	2021	20‐05‐2021 04:33	10‐10‐2021 14:54	14	61	0.345208	0.002065
Great Black‐backed Gull	1750	2021	2021	14‐05‐2021 07:30	27‐08‐2021 10:32	100	1218	0.129787	4.94E−05
Great Black‐backed Gull	1751	2021	2021	17‐05‐2021 10:35	06‐08‐2021 12:53	6	23	0.121677	2.26E−06
Great Black‐backed Gull	1752	2021	2021	20‐05‐2021 20:03	27‐08‐2021 18:29	25	318	0.300922	0.000465
Great Black‐backed Gull	1755	2021	2021	19‐05‐2021 10:03	26‐08‐2021 14:23	9	68	0.857031	0.002648
Herring Gull	1399	2019	2019	27‐06‐2019 08:24	17‐08‐2019 18:51	35	556	na	na
Herring Gull	1402	2019	2019	17‐07‐2019 19:40	23‐08‐2019 17:04	38	595	na	na
Herring Gull	1402	2019	2020	29‐03‐2020 20:33	05‐08‐2020 20:00	29	400	na	na
Herring Gull	1600	2021	2021	24‐05‐2021 06:49	13‐08‐2021 19:29	62	639	0.870798	0.000353
Herring Gull	1617	2021	2021	23‐05‐2021 13:48	27‐08‐2021 11:57	87	670	0.671215	0.000374
Herring Gull	1618	2021	2021	22‐05‐2021 12:06	10‐10‐2021 14:52	97	1254	0.184196	4.66E−05
Herring Gull	1621	2021	2021	23‐05‐2021 06:52	10‐10‐2021 14:43	96	1117	0.896876	0.000176
Herring Gull	1622	2021	2021	06‐07‐2021 11:28	18‐08‐2021 08:43	43	687	0.677155	0.00035
Herring Gull	1623	2021	2021	23‐05‐2021 14:11	08‐08‐2021 19:21	70	633	0.720574	0.000397
Herring Gull	1624	2021	2021	01‐06‐2021 06:47	27‐08‐2021 15:10	88	1027	0.723936	0.000242
Herring Gull	1625	2021	2021	01‐06‐2021 09:22	30‐07‐2021 12:22	57	514	0.404892	0.000413
Herring Gull	1627	2021	2021	01‐06‐2021 10:09	27‐08‐2021 15:03	87	1433	0.832702	4.61E−05
Herring Gull	1629	2021	2021	31‐05‐2021 13:52	10‐10‐2021 14:30	65	757	0.819167	0.000281
Herring Gull	1630	2021	2021	23‐05‐2021 07:58	27‐08‐2021 12:40	93	954	0.405441	0.000197
Herring Gull	1632	2021	2021	23‐05‐2021 14:24	27‐08‐2021 10:58	83	759	0.731844	0.00028
Herring Gull	1617	2021	2022	28‐04‐2022 08:51	10‐08‐2022 11:23	83	448	na	na
Herring Gull	1618	2021	2022	27‐04‐2022 04:34	15‐09‐2022 21:33	111	1156	na	na
Herring Gull	1621	2021	2022	27‐04‐2022 05:19	15‐09‐2022 12:04	103	1763	na	na
Herring Gull	1622	2021	2022	27‐04‐2022 11:50	13‐08‐2022 06:51	78	459	na	na
Herring Gull	1623	2021	2022	27‐04‐2022 10:53	13‐09‐2022 11:22	98	993	na	na
Herring Gull	1627	2021	2022	27‐04‐2022 06:00	15‐09‐2022 12:19	141	2182	na	na
Herring Gull	1629	2021	2022	28‐04‐2022 10:23	24‐06‐2022 15:12	50	596	na	na
Herring Gull	1630	2021	2022	27‐04‐2022 04:21	15‐09‐2022 11:34	136	1702	na	na
Herring Gull	1632	2021	2022	27‐04‐2022 04:49	19‐08‐2022 11:43	91	1188	na	na
Herring Gull	1617	2021	2023	07‐04‐2023 05:39	26‐08‐2023 09:56	119	627	na	na
Herring Gull	1621	2021	2023	09‐03‐2023 14:02	01‐07‐2023 10:53	8	8	na	na
Herring Gull	1623	2021	2023	07‐04‐2023 06:33	19‐07‐2023 01:29	71	335	na	na
Herring Gull	1627	2021	2023	07‐04‐2023 12:13	03‐09‐2023 12:20	147	2174	na	na
Herring Gull	1629	2021	2023	08‐03‐2023 12:57	18‐06‐2023 16:32	60	269	na	na
Herring Gull	1630	2021	2023	06‐04‐2023 22:03	29‐04‐2023 05:50	21	308	na	na
Herring Gull	1632	2021	2023	07‐04‐2023 11:17	04‐10‐2023 00:48	132	1340	na	na
Lesser Black‐backed Gull	1126	2019	2019	27‐06‐2019 07:09	05‐08‐2019 16:41	38	214	na	na
Lesser Black‐backed Gull	1145	2019	2019	27‐06‐2019 16:43	22‐08‐2019 18:09	55	830	na	na
Lesser Black‐backed Gull	1167	2019	2019	28‐06‐2019 04:49	29‐08‐2019 17:08	61	461	na	na
Lesser Black‐backed Gull	5824	2019	2019	04‐06‐2019 10:56	16‐07‐2019 21:27	42	742	na	na
Lesser Black‐backed Gull	5827	2019	2019	05‐06‐2019 11:11	10‐08‐2019 19:31	64	1243	na	na
Lesser Black‐backed Gull	5828	2019	2019	04‐06‐2019 03:37	01‐08‐2019 20:08	59	1130	na	na
Lesser Black‐backed Gull	5829	2019	2019	02‐06‐2019 08:58	15‐07‐2019 06:00	44	536	na	na
Lesser Black‐backed Gull	5841	2019	2019	24‐05‐2019 14:32	29‐07‐2019 18:35	61	1011	na	na
Lesser Black‐backed Gull	5842	2019	2019	04‐06‐2019 02:42	24‐08‐2019 17:42	81	1396	na	na
Lesser Black‐backed Gull	5844	2019	2019	27‐05‐2019 04:51	29‐07‐2019 19:53	56	1362	na	na
Lesser Black‐backed Gull	5845	2019	2019	25‐05‐2019 02:38	22‐08‐2019 19:07	89	1413	na	na
Lesser Black‐backed Gull	5846	2019	2019	25‐05‐2019 03:24	06‐08‐2019 19:54	68	1362	na	na
Lesser Black‐backed Gull	5847	2019	2019	03‐06‐2019 14:29	23‐08‐2019 13:49	78	1385	na	na
Lesser Black‐backed Gull	5848	2019	2019	03‐06‐2019 12:17	08‐08‐2019 05:21	60	1010	na	na
Lesser Black‐backed Gull	5849	2019	2019	06‐06‐2019 04:16	10‐07‐2019 20:25	33	483	na	na
Lesser Black‐backed Gull	5850	2019	2019	25‐05‐2019 10:28	12‐08‐2019 21:30	76	2003	na	na
Lesser Black‐backed Gull	5851	2019	2019	04‐06‐2019 08:01	24‐08‐2019 19:01	81	1276	na	na
Lesser Black‐backed Gull	5852	2019	2019	24‐05‐2019 23:25	17‐08‐2019 19:23	83	1210	na	na
Lesser Black‐backed Gull	5854	2019	2019	02‐06‐2019 03:08	09‐08‐2019 16:06	66	912	na	na
Lesser Black‐backed Gull	5855	2019	2019	02‐06‐2019 22:43	18‐07‐2019 01:13	36	1034	na	na
Lesser Black‐backed Gull	5856	2019	2019	24‐05‐2019 00:16	08‐08‐2019 20:34	77	1426	na	na
Lesser Black‐backed Gull	5857	2019	2019	03‐06‐2019 13:37	28‐06‐2019 20:38	26	928	na	na
Lesser Black‐backed Gull	5858	2019	2019	05‐06‐2019 02:17	18‐08‐2019 19:18	45	468	na	na
Lesser Black‐backed Gull	5859	2019	2019	03‐06‐2019 15:20	24‐08‐2019 18:19	77	1480	na	na
Lesser Black‐backed Gull	5894	2019	2019	26‐05‐2019 10:14	08‐08‐2019 15:42	69	824	na	na
Lesser Black‐backed Gull	5957	2019	2019	02‐06‐2019 08:19	09‐06‐2019 19:49	8	167	na	na
Lesser Black‐backed Gull	5958	2019	2019	02‐06‐2019 11:09	07‐09‐2019 17:40	82	2323	na	na
Lesser Black‐backed Gull	5959	2019	2019	04‐06‐2019 04:05	11‐08‐2019 20:01	68	1442	na	na
Lesser Black‐backed Gull	1126	2019	2020	01‐05‐2020 16:09	14‐07‐2020 06:57	59	259	na	na
Lesser Black‐backed Gull	1145	2019	2020	25‐04‐2020 05:16	06‐08‐2020 17:13	96	1821	na	na
Lesser Black‐backed Gull	1167	2019	2020	09‐05‐2020 19:47	04‐08‐2020 19:44	73	279	na	na
Lesser Black‐backed Gull	5827	2019	2020	12‐06‐2020 14:39	30‐08‐2020 11:35	50	1557	na	na
Lesser Black‐backed Gull	5828	2019	2020	12‐06‐2020 17:39	24‐06‐2020 12:43	13	430	na	na
Lesser Black‐backed Gull	5842	2019	2020	19‐06‐2020 03:56	01‐08‐2020 20:27	33	1067	na	na
Lesser Black‐backed Gull	5845	2019	2020	20‐03‐2020 07:53	09‐08‐2020 20:54	64	1341	na	na
Lesser Black‐backed Gull	5847	2019	2020	16‐06‐2020 06:53	15‐07‐2020 16:23	25	634	na	na
Lesser Black‐backed Gull	5848	2019	2020	23‐03‐2020 09:00	26‐06‐2020 19:01	18	391	na	na
Lesser Black‐backed Gull	5849	2019	2020	03‐04‐2020 09:53	29‐06‐2020 18:54	25	447	na	na
Lesser Black‐backed Gull	5850	2019	2020	17‐06‐2020 12:25	03‐08‐2020 19:52	27	1059	na	na
Lesser Black‐backed Gull	5851	2019	2020	18‐06‐2020 11:58	29‐07‐2020 20:01	30	718	na	na
Lesser Black‐backed Gull	5852	2019	2020	24‐03‐2020 11:43	23‐07‐2020 16:14	42	814	na	na
Lesser Black‐backed Gull	5854	2019	2020	12‐06‐2020 03:01	10‐08‐2020 18:52	57	1064	na	na
Lesser Black‐backed Gull	5856	2019	2020	12‐06‐2020 04:13	08‐07‐2020 19:10	25	871	na	na
Lesser Black‐backed Gull	5858	2019	2020	17‐06‐2020 10:54	06‐08‐2020 19:25	10	83	na	na
Lesser Black‐backed Gull	5859	2019	2020	13‐06‐2020 07:32	19‐07‐2020 19:33	11	198	na	na
Lesser Black‐backed Gull	5894	2019	2020	27‐06‐2020 03:48	19‐08‐2020 17:12	49	894	na	na
Lesser Black‐backed Gull	5958	2019	2020	30‐07‐2020 04:09	10‐08‐2020 20:39	12	409	na	na
Lesser Black‐backed Gull	5959	2019	2020	17‐06‐2020 12:39	24‐06‐2020 20:10	8	281	na	na
Lesser Black‐backed Gull	1126	2019	2021	25‐04‐2021 05:19	15‐08‐2021 05:44	87	461	0.583582	0.000544
Lesser Black‐backed Gull	1129	2021	2021	04‐06‐2021 12:36	22‐08‐2021 20:11	78	872	0.784311	0.000111
Lesser Black‐backed Gull	1131	2021	2021	08‐06‐2021 11:23	26‐08‐2021 19:18	48	370	0.756775	0.000194
Lesser Black‐backed Gull	1132	2021	2021	06‐06‐2021 06:51	06‐08‐2021 19:17	45	231	0.823383	0.000451
Lesser Black‐backed Gull	1135	2021	2021	05‐06‐2021 06:24	15‐08‐2021 18:54	64	547	0.800972	0.000382
Lesser Black‐backed Gull	1137	2021	2021	05‐06‐2021 05:10	18‐08‐2021 18:44	66	383	0.897052	0.000483
Lesser Black‐backed Gull	1142	2021	2021	04‐06‐2021 16:59	18‐08‐2021 18:42	73	529	0.751972	0.000407
Lesser Black‐backed Gull	1143	2021	2021	03‐06‐2021 14:36	19‐07‐2021 19:50	44	296	0.527082	0.000817
Lesser Black‐backed Gull	1145	2019	2021	27‐04‐2021 06:09	25‐07‐2021 20:38	85	1222	0.764403	2.75E−05
Lesser Black‐backed Gull	1163	2021	2021	04‐06‐2021 13:48	15‐08‐2021 18:10	71	560	0.791486	0.000152
Lesser Black‐backed Gull	1166	2021	2021	04‐06‐2021 07:53	11‐07‐2021 09:03	12	55	0.697164	0.000942
Lesser Black‐backed Gull	1167	2019	2021	08‐05‐2021 14:40	27‐08‐2021 08:52	77	266	0.8289	0.000113
Lesser Black‐backed Gull	1170	2021	2021	04‐06‐2021 06:26	05‐08‐2021 18:48	62	613	0.85565	0.000139
Lesser Black‐backed Gull	1173	2021	2021	04‐06‐2021 11:03	06‐07‐2021 20:33	28	318	0.511371	0.000765
Lesser Black‐backed Gull	1174	2021	2021	05‐06‐2021 08:54	13‐08‐2021 19:33	22	86	0.826377	0.000873
Lesser Black‐backed Gull	5827	2019	2021	18‐04‐2021 07:42	22‐08‐2021 19:38	122	2206	0.763199	5.29E−05
Lesser Black‐backed Gull	5828	2019	2021	01‐04‐2021 06:08	12‐07‐2021 19:36	96	2242	0.762151	1.59E−05
Lesser Black‐backed Gull	5842	2019	2021	20‐04‐2021 05:12	03‐08‐2021 15:16	86	1300	0.808021	5.62E−05
Lesser Black‐backed Gull	5845	2019	2021	22‐03‐2021 06:16	06‐08‐2021 19:42	124	2639	0.787236	2.29E−05
Lesser Black‐backed Gull	5846	2019	2021	03‐04‐2021 06:24	23‐07‐2021 05:21	88	2659	0.771423	1.73E−05
Lesser Black‐backed Gull	5848	2019	2021	14‐04‐2021 15:56	06‐06‐2021 10:58	12	135	0.771981	0.000268
Lesser Black‐backed Gull	5849	2019	2021	01‐04‐2021 05:37	30‐07‐2021 15:06	22	358	0.767953	0.000165
Lesser Black‐backed Gull	5850	2019	2021	01‐05‐2021 12:55	07‐08‐2021 18:55	89	2596	0.836961	1.75E−05
Lesser Black‐backed Gull	5851	2019	2021	10‐04‐2021 12:16	03‐08‐2021 20:49	84	1919	0.811098	2.38E−05
Lesser Black‐backed Gull	5852	2019	2021	27‐03‐2021 06:37	21‐08‐2021 17:42	137	2288	0.794384	2.86E−05
Lesser Black‐backed Gull	5854	2019	2021	17‐04‐2021 10:14	26‐08‐2021 19:15	100	1228	0.823395	0.000185
Lesser Black‐backed Gull	5856	2019	2021	06‐05‐2021 11:35	10‐08‐2021 14:04	53	624	0.785026	0.00012
Lesser Black‐backed Gull	5858	2019	2021	02‐04‐2021 10:30	29‐07‐2021 20:57	65	1114	0.787008	4.84E−05
Lesser Black‐backed Gull	5859	2019	2021	28‐03‐2021 06:48	14‐04‐2021 16:46	4	31	na	na
Lesser Black‐backed Gull	5894	2019	2021	13‐04‐2021 08:51	26‐07‐2021 20:21	96	2594	0.846285	2.97E−05
Lesser Black‐backed Gull	5958	2019	2021	16‐04‐2021 05:57	02‐08‐2021 20:25	105	2492	0.824478	4.61E−05
Lesser Black‐backed Gull	1129	2021	2022	27‐04‐2022 04:26	17‐07‐2022 12:06	69	630	na	na
Lesser Black‐backed Gull	1132	2021	2022	27‐04‐2022 05:21	03‐08‐2022 10:13	76	425	na	na
Lesser Black‐backed Gull	1135	2021	2022	27‐04‐2022 05:21	02‐05‐2022 09:37	6	50	na	na
Lesser Black‐backed Gull	1137	2021	2022	27‐04‐2022 05:33	18‐08‐2022 12:23	92	595	na	na
Lesser Black‐backed Gull	1142	2021	2022	29‐04‐2022 06:07	11‐06‐2022 11:39	9	56	na	na
Lesser Black‐backed Gull	1163	2021	2022	27‐04‐2022 10:52	16‐08‐2022 22:47	80	593	na	na
Lesser Black‐backed Gull	1166	2021	2022	26‐04‐2022 23:53	05‐08‐2022 04:34	60	331	na	na
Lesser Black‐backed Gull	1170	2021	2022	27‐04‐2022 04:57	27‐06‐2022 21:53	59	811	na	na
Lesser Black‐backed Gull	5827	2019	2022	06‐05‐2022 04:35	16‐06‐2022 17:05	37	650	na	na
Lesser Black‐backed Gull	5845	2019	2022	26‐03‐2022 10:22	13‐08‐2022 19:51	114	2405	na	na
Lesser Black‐backed Gull	5846	2019	2022	06‐04‐2022 15:43	16‐06‐2022 04:46	53	1268	na	na
Lesser Black‐backed Gull	5848	2019	2022	05‐04‐2022 05:51	14‐06‐2022 01:57	14	225	na	na
Lesser Black‐backed Gull	5849	2019	2022	20‐06‐2022 16:05	19‐07‐2022 07:37	10	79	na	na
Lesser Black‐backed Gull	5850	2019	2022	08‐05‐2022 12:22	07‐07‐2022 19:48	34	339	na	na
Lesser Black‐backed Gull	5851	2019	2022	25‐05‐2022 17:33	11‐07‐2022 20:25	7	120	na	na
Lesser Black‐backed Gull	5852	2019	2022	27‐03‐2022 10:19	08‐04‐2022 17:51	9	75	na	na
Lesser Black‐backed Gull	5854	2019	2022	26‐03‐2022 13:18	17‐08‐2022 15:58	103	1105	na	na
Lesser Black‐backed Gull	5858	2019	2022	31‐03‐2022 10:06	04‐08‐2022 05:14	77	1117	na	na
Lesser Black‐backed Gull	1129	2021	2023	07‐04‐2023 11:45	03‐08‐2023 11:54	103	949	na	na
Lesser Black‐backed Gull	1137	2021	2023	10‐04‐2023 05:25	13‐08‐2023 12:19	75	596	na	na
Lesser Black‐backed Gull	1163	2021	2023	14‐04‐2023 11:41	26‐08‐2023 12:00	43	163	na	na
Lesser Black‐backed Gull	1166	2021	2023	09‐04‐2023 05:29	10‐08‐2023 05:15	81	466	na	na
Lesser Black‐backed Gull	1174	2021	2023	07‐07‐2023 04:20	30‐07‐2023 09:45	7	27	na	na
